# Direct observations of melting, freezing, and ocean circulation in an ice shelf basal crevasse

**DOI:** 10.1126/sciadv.adi7638

**Published:** 2023-10-27

**Authors:** Peter Washam, Justin D. Lawrence, Craig L. Stevens, Christina L. Hulbe, Huw J. Horgan, Natalie J. Robinson, Craig L. Stewart, Anthony Spears, Enrica Quartini, Benjamin Hurwitz, Matthew R. Meister, Andrew D. Mullen, Daniel J. Dichek, Frances Bryson, Britney E. Schmidt

**Affiliations:** ^1^ Department of Astronomy, Cornell University, Ithaca, NY, USA.; ^2^ School of Earth and Atmospheric Sciences, Georgia Institute of Technology, Atlanta, GA, USA.; ^3^ Honeybee Robotics, Altadena, CA, USA.; ^4^ Ocean Dynamics Group, National Institute for Water and Atmospheric Research, Greta Point, Wellington, New Zealand.; ^5^ Department of Physics, University of Auckland, Auckland, New Zealand.; ^6^ School of Surveying, University of Otago, Dunedin, New Zealand.; ^7^ Laboratory of Hydraulics, Hydrology and Glaciology (VAW), ETH Zurich, Zurich, Switzerland.; ^8^ Swiss Federal Institute for Forest, Snow, and Landscape Research (WSL), Birmensdorf, Switzerland.; ^9^ Antarctic Research Centre, Victoria University of Wellington, Wellington, New Zealand.

## Abstract

Ocean conditions near the grounding zones of Antarctica’s ice shelves play a key role in controlling the outflow and mass balance of the ice sheet. However, ocean observations in these regions are largely absent. Here, we present a detailed spatial survey collected with an underwater vehicle in a basal crevasse located in the ocean cavity at the Ross Ice Shelf grounding zone. The observations depict fine-scale variability in ocean forcing that drives asymmetric melting along the lower crevasse sidewalls and freezing in the upper reaches of the crevasse. Freshwater release from melting at depth and salt rejection from freezing above drives an overturning circulation. This vertical circulation pattern overlays a dominant throughflow jet, which funnels water parallel to the coastline, orthogonal to the direction of tidal currents. Importantly, these data reveal that basal crevasses influence ocean circulation and mixing at ice shelf grounding zones to an extent previously unknown.

## Teaser

In situ observations reveal how ocean physical properties drive ice melting and freezing within an ice shelf basal crevasse.

## INTRODUCTION

Ice shelves form around the coastline of Antarctica where the ice sheet detaches from the underlying bed to float on the ocean. They occupy a ∼1.6 million–km^2^ area of the surrounding coastal ocean and comprise 11% of the combined ice sheet and ice shelf area (*1*). Ice shelves play a key role in the mass balance of the Antarctic Ice Sheet, as they not only facilitate the majority of its mass loss through ocean-driven melting and calving (*2*) but also modulate the flux of ice into the ocean through resistive buttressing forces (*3*). Several large Antarctic ice shelves have thinned and retreated, or completely disintegrated in recent years, followed by notable increases in the seaward flow of the upstream glaciers (*4*–*6*). This process is presently most important in the Amundsen Sea sector of West Antarctica, where increased ice discharge attributed to ice shelf changes constituted 0.8 cm of the 1.4 ± 0.2 cm that Antarctica contributed to global mean sea level rise between 1979 and 2017 (*2*).

Future mass loss from the Antarctic Ice Sheet is projected to increase to contribute 6 to 53 cm to the global mean sea level rise by 2100, given anthropogenic climate change scenarios between 1.5° and 5°C warming above preindustrial levels (*7*). A major source of uncertainty in these projections is how ice shelf changes, predominantly related to ice-ocean interactions (*8*), will translate upstream to influence grounded ice sheet dynamics (*7*). This is due to the complex timing surrounding the onset and evolution of dynamic instabilities in the ice shelf–ice sheet system (*8*) and present unknowns related to these instabilities (*7*). However, the global climate models used to represent ocean conditions in circum-Antarctic Ice Sheet models require very coarse grid sizes of several tens of thousands of meters in the horizontal and greater than 10 m in the vertical (*9*). Regional models capable of simulating coupled interactions between a single glacier catchment, ice shelf, and ocean have somewhat finer horizontal and vertical resolutions of several thousand meters and 10 m, respectively (*10*). Nonetheless, models of these scales must parameterize ocean processes that exist on smaller spatial scales than their grid sizes, as well as small-scale variations in ice slope (*11*) and surface shape (*12*–*14*) that interact with the ocean in a coupled manner. These smaller scales can profoundly influence localized ice shelf melting, marine ice formation, and the coupled oceanic circulation (*15*, *16*), which in turn affects the larger ice shelf’s integrity. Therefore, correct parameterization of these processes is key to accurately projecting Antarctica’s future contribution to sea level rise.

Very few direct observations of the ocean cavity below ice shelves exist to constrain the hydrographic properties and parameterized physical processes simulated in ice sheet models. This is because of the intense logistical undertaking required to access the ocean cavity, either by drilling through the ice shelf (*17*) or by deploying mobile underwater vehicles from a ship at the ice shelf calving front that are capable of navigating underneath it (*18*). The scarcity of sub–ice shelf ocean observations includes the region just downstream of the point where ice transitions from resting on the underlying bed to first encounter the ocean, referred to as the grounding line (GL). Throughout the text, we refer to the region spanning from the furthest tidally affected inland GL location to the point of full hydrostatic flotation as the grounding zone (GZ). To date, published oceanographic measurements exist in less than 10 Antarctic GZ ocean cavities (*19*–*25*). This leaves much uncertainty about the hydrographic properties, circulation patterns, and dominant scales of variability that drive ice-ocean interactions in these regions around Antarctica. This is a critical observational shortcoming because variations in ocean forcing contact the ice shelf at its thickest point here where buttressing forces are generally at a maximum (*26*).

The ice in the GZ undergoes a dynamic adjustment as it transitions from grounded to hydrostatic flotation, which creates stress in the ice column that can propagate crevasses from either the surface or base (*27*, *28*). Once initiated, the shape of basal crevasses changes over time due to viscous deformation of the surrounding ice column and thermodynamic interaction with the underlying ocean (*29*). Basal crevasses can either close as they advect downstream into lower stress regions of the ice shelf or widen as a result of increased forcing from the above viscous processes or from elastic processes (*29*). If basal crevasses do grow sufficiently wide, then they can propagate through the entire ice column, producing rifts that further reduce ice shelf integrity. It is estimated that pervasive rifting leading to iceberg calving removes the majority of mass from the Filchner-Ronne, Ross, and Amery ice shelves (*30*), Antarctica’s three largest ice shelves that constitute 63% of the total ice shelf area (*1*). There are only four published studies that present in situ ocean measurements from within a crevasse or rift (*20*, *31*–*33*), none of which discuss three-dimensional circulation and detailed melt and freeze rates throughout the feature. This results in large uncertainty about the ocean’s role in crevasse evolution and ice shelf stability and how crevasses in turn influence ocean cavity circulation.

The Filchner-Ronne, Ross, and Amery ice shelves have been stable over the modern satellite observational record (*2*), in large part because they overlie cold ocean cavities that are flooded with a water mass, which forms when salt is rejected during atmospherically driven winter sea ice formation in the polynyas that fringe their calving fronts (*34*–*38*). This water mass, referred to as high-salinity shelf water (HSSW), has hydrographic properties consistent with the surface freezing point of seawater: thermal driving (Θ − Θ_f_) = 0°C, absolute salinity (*S*_A_) ≥ 34.67 g kg^−1^, and density anomaly (σ_Θ_) ≥ 27.78 kg m^−3^ (*34*, *36*, *37*). While this dense water mass contains no sensible heat at the surface, as it sinks below the ice, the increased pressure at depth reduces the freezing point of seawater, raising its conservative temperature (Θ) above the freezing point and imparting a positive thermal driving (Θ − Θ_f_ *>* 0°C), which allows it to melt ice. Note that hereafter we simply refer to conservative temperature as temperature and absolute salinity as salinity.

When seawater drives melt along the ice base, it converts its sensible heat into the latent heat required to change solid glacial ice into liquid freshwater with a 0 g kg^−1^ salinity. This phase change expends considerable heat, which gives the resultant glacial meltwater (GMW) an effective temperature of −92.50°C (*39*). Turbulent mixing quickly dilutes the GMW into the underlying seawater to concentrations on the order of several milliliters per liter in cold sub–ice shelf ocean cavities (*40*–*42*). Nevertheless, when the water column is sufficiently weakly stratified and the ice base slopes upward, the lower density of the freshened glacially modified water mass mixture causes it to rise along the ice base to lower pressures. In the low-temperature environments of cold ocean cavity ice shelves, this temperature reduction, admixture of freshwater, and pressure reduction leads to supercooling (Θ − Θ_f_ *<* 0°C) in the water column that forms saline marine ice with a temperature characteristic of the salinity- and pressure-dependent freezing point (*43*).

Marine ice forms as either small frazil crystals, typically *<* 0.10 cm in diameter (*44*–*46*), suspended in the water column or through direct freezing onto the ice base. While the relative contribution of these two formation mechanisms is not well constrained, their combined effect results in marine ice layers of various porosity that accumulate on ice shelf undersides in thicknesses up to hundreds of meters (*47*, *48*), which alter ice shelf flow and promote ice shelf stability through infill of basal features such as crevasses (*49*–*51*). This sequence of ice-ocean thermodynamic interactions that removes glacial ice at greater depths and deposits marine ice along shallower portions of the ice shelf is referred to as the “ice pump” (*52*).

**Fig. 1.**
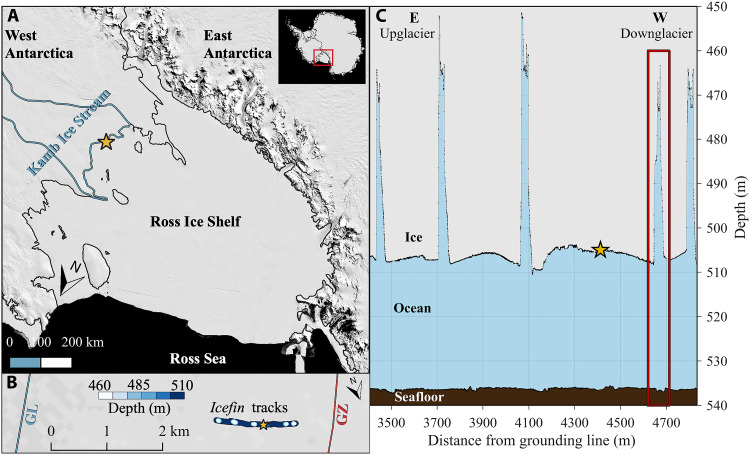
Geographic location of the study site. (**A**) Moderate Resolution Imaging Spectroradiometer (MODIS) mosaic (*79*) of RIS, which lies at the boundary of East and West Antarctica. The black line delineates these two ice sheets and represents the GL of RIS from (*55*). The gold star indicates the drill site and the light blue line highlights the stagnant Kamb Ice Stream margins. The inset positions RIS relative to the Antarctic continent. (**B**) Map view of the GZ region just downstream of the RIS GL where the *Icefin* survey was collected. The measured ice base is colored by depth, the gold star denotes the drill site, and the red line indicates the outer GZ boundary from (*56*). (**C**) Cross-sectional view of ice, ocean, and seafloor geometry from the *Icefin* survey (composite from dive 2 and 3 data); the gold star highlights the borehole location and the red box outlines the crevasse principally studied in this text. Note the vertical scale exaggeration in (C).

Here, we present observational spatial survey data from a basal crevasse located in an Antarctic GZ ocean cavity. These data were collected with the remotely operated underwater vehicle *Icefin* (*20*, *24*) beneath the Ross Ice Shelf (RIS), seaward of the Kamb Ice Stream GL (Fig. 1). They are an extension of those discussed in (*20*), which described ice, ocean, and seafloor properties over the full region surveyed with *Icefin*. This survey was made possible by deploying *Icefin* through a borehole drilled with hot water. The lower trunk of Kamb Ice Stream near its GL stagnated around 1860 (*53*), likely due to a diversion of lubricating subglacial water to neighboring ice streams (*54*) that actively flow into RIS at up to 800 m year^−1^ (*2*). The ice in our study region is 520 to 580 m thick, lies at the outer edge of the GZ at 3400 to 4900 m seaward of the GL (Fig. 1) (*55*, *56*), oscillates vertically with the tide by up to 1.50 m in near-perfect hydrostatic flotation (*56*), and advances very slowly at ∼5 m year^−1^ (*2*). The cold ocean conditions at the site paired with this near lack of ice advection make it a useful opportunity for a very detailed investigation of small-scale ocean processes driving melting and freezing in an ice shelf basal crevasse. The results describe ocean circulation and ice-ocean interactions within a crevasse in unprecedented detail and provide necessary constraints on ocean conditions and parameterizations used to simulate ice-ocean thermodynamics in ice sheet models.

## RESULTS

### Geometry of basal crevasses

**Fig. 2.**
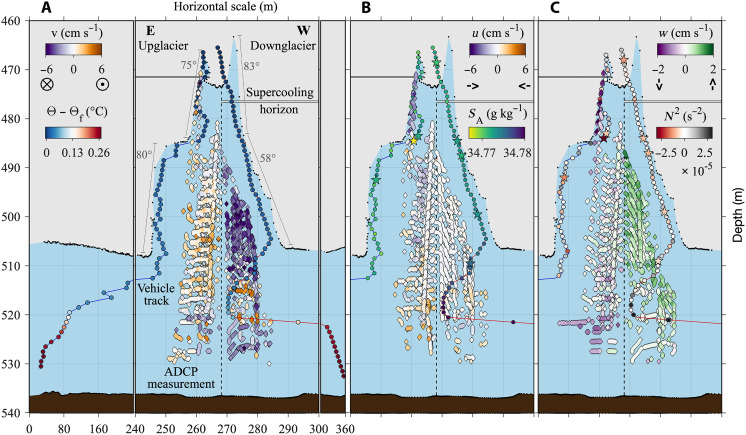
Three-dimensional ocean circulation and hydrography in the crevasse. (**A**) ADCP measurements (diamond symbols) colored by the *v* velocity component, where positive values indicate northward direction (out of the page) and negative values denote southward direction (into the page). Points along the vehicle tracks of the red line (western) and blue line (eastern) represent hydrographic bin locations (Materials and Methods) and are colored by thermal driving (Θ − Θ_f_). (**B** and **C**) As in (A), except only the crevasse region at horizontal distances of 240 to 300 m. In (B), ADCP measurements are colored by the *u* velocity component, where positive is eastward and negative is westward, and hydrographic bins are colored by salinity (*S*_A_). In (C), ADCP measurements are colored by the *w* velocity component, where positive is upward and negative is downward, and hydrographic bins are colored by stratification frequency (*N*^2^). Note that the color scale represents a different velocity range in (C). Stars in (B) and (C) highlight vertical salinity (and therefore density) variations that result in stratification frequencies significantly below 0 s^−2^, considering the ±1 SD range. The vertical dashed line delineates the western and eastern section of the crevasse based on distinctive circulation patterns, the horizontal white lines mark the supercooling horizon in each profile, and lines parallel to crevasse walls in (A) indicate their varying slopes. Additional black points on the ice base and seafloor are elevation locations sampled by *Icefin*’s altimeter and DVL.

The ∼1500-m-long spatial survey beneath RIS identified five crevasses with the laser altimeter onboard *Icefin* (Materials and Methods). These features extended upward from a relatively flat ice shelf base with concave features separating the crevasses (Fig. 1C). An ocean cavity underlaid the ice, ranging from ∼30-m thickness beneath the base to ∼80 m beneath the crevasse roofs, where seafloor elevations were measured with the vehicle’s Doppler velocity logger. The flat seafloor exhibited 0.50-m-tall ridges that were horizontally offset from the crevasses by ∼250 m [see (*20*) for further description on the ocean cavity and seafloor]. The crevasses were generally oriented parallel to the GL in the north-south direction (Fig. 1B), were trapezoidal in shape, ranged from 43 to 59 m in height and from 42 to 56 m in width at their widest point, and exhibited a slight asymmetry with a shallower slope along their western (downglacier) wall at depths below 485 m (Fig. 1C). The crevasse examined in detail with *Icefin* was 45 m tall and 45 m wide at its base, narrowing to ∼10 m at the roof, with noticeably different sidewall geometries (Fig. 2). The eastern (upglacier) wall exhibited a 10-m-wide flat, step-like feature at around 485-m depth that separated the wall into an upper and lower face with respective slopes of 75° and 80°. The other four crevasses mapped in the under-ice survey did not have a step-like feature along their eastern wall. This suggests that it was either unique to the crevasse studied in detail or a result of sampling a three-dimensional morphological feature incised in the crevasse wall in only two dimensions. The western wall was instead a continuous ice face that transitioned from a steeper slope of 83° above 485-m depth to a notably more gradual slope of 58° below. Note that ice base elevations presented here come from the main, outbound portion of the dive and the oceanographic data from the return leg (fig. S1). Positional discrepancies of hydrographic data within the crevasse result from variation in its geometry between the locations sampled on the outbound and return dive sections (see Materials and Methods for further discussion).

### Ocean circulation and ice-ocean interactions within the crevasse

Ocean velocities were recorded with a downward-facing acoustic Doppler current profiler onboard *Icefin* (Materials and Methods). The ocean circulation within the crevasse exhibited a three-dimensional structure that varied considerably with horizontal and vertical position in the feature. The strongest velocities flowed southward (parallel to the GL) through the crevasse at a moderate rate of *v* = −6 cm s^−1^ in a jet concentrated along the western sidewall below 490-m depth (Fig. 2A). An overturning circulation superimposed this prevailing throughflow, with rising in the western half of the crevasse and sinking in the eastern half at rates up to |*w*| = 2 cm s^−1^ (Fig. 2C). The downwelling was concentrated in a plume along the eastern wall at depths shallower than 485 m that also flowed westward across the crevasse at *u* = −4 cm s^−1^ (Fig. 2B). The east-west velocity component outside of the downwelling plume was generally weak with no distinguishable pattern. Ocean circulation beneath the crevasse base behaved similarly to within the feature, except for a noisy region from 515- to 525-m depth where flow reversals occurred (fig. S2). This variability centered on the sharp pycnocline (fig. S3, A to C), implying internal wave activity, small-scale mixing within the pycnocline, or baroclinic flow patterns associated with larger-scale density variations (*57*).

**Fig. 3.**
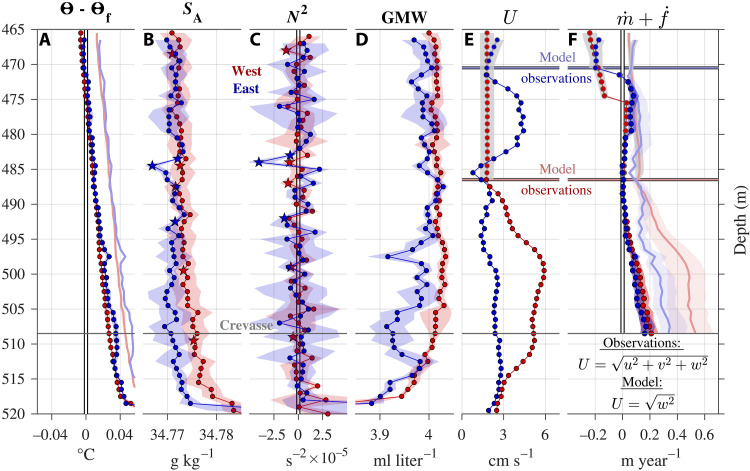
Vertical profiles of ocean properties and ice-ocean thermodynamics in the crevasse. (**A**) Ocean temperature relative to freezing (i.e., thermal driving), (**B**) salinity, (**C**) stratification frequency (*N*^2^ = −*g/*ρ_0_ δρ*/*δ*z*), (**D**) glacial meltwater concentration (Materials and Methods), (**E**) current speed, and (**F**) ice surface ($\dot{m}$) and frazil ($\dot{f}$) melt or freeze rate (Materials and Methods) on the west (red) and east (blue) halves of the crevasse. Data plotted in (A) to (E) represent 1-m bin averages of in situ hydrographic data recorded by *Icefin,* and the background shading is the ±1 SD range (Materials and Methods). Stars in (B) and (C) highlight vertical salinity (and therefore density) variations that result in stratification frequencies significantly below 0 s^−2^, considering the ±1 SD range. Red and blue lines in (E) to (F) delineate between observations and model data on the west and east halves of the crevasse, respectively. Observed current speeds are the magnitude of the three-dimensional velocity components closest to the crevasse wall at each binned depth and the modeled current speeds represent only the magnitude of the vertical velocity component (Materials and Methods). Light red and blue data in (A) and (F) represent predicted conditions during spring tide (Materials and Methods). Red and blue shaded data in (F) are the uncertainty ranges associated with melt and freeze rates (Materials and Methods). Gray-shaded data in (E) are the modeled vertical velocity component and in (F) the melt or freeze rate (Materials and Methods). Horizontal gray lines demarcate the crevasse base and roof, and the vertical white lines highlight zero values.

**Fig. 4.**
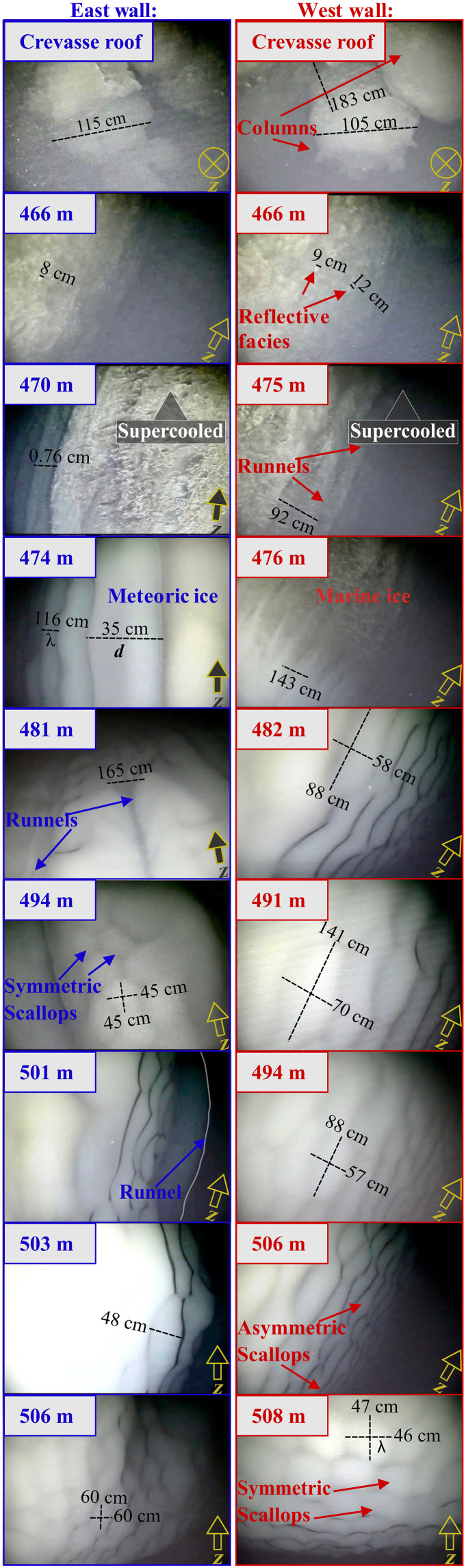
Ice morphology present in the crevasse walls. Images taken with *Icefin*’s forward looking camera at various depths along the east (blue outline) and west (red outline) crevasse walls. Images at depths above 471 m (east) and 475 m (west) are within the supercooled portion of the crevasse. The scale of features was measured with *Icefin*’s forward-looking sonar and is listed in table S1. The wavelength (λ) and incision depth (*d*) of select features are indicated. Arrows in the bottom right of each panel align the image with the vertical. Gray lines outline a runnel in the background of the image taken at 501-m depth along the eastern sidewall. Video footage of the crevasse sidewalls can be found in the Supplementary Materials of (*20*).

The conductivity-temperature and pressure sensors on *Icefin* provided hydrographic conditions in close proximity to the ice surface along each crevasse sidewall (Fig. 2; Materials and Methods). Vertically bin-averaged profiles of the hydrography inside the crevasse captured the buoyancy dynamics resulting from ice melting and freezing (Fig. 3) that drove the observed overturning circulation (Fig. 2). Ocean temperatures transitioned from above freezing at the crevasse base to below freezing in the upper crevasse at 475- and 471-m depth or 10 and 5 m below the roof on the western and eastern half, respectively (Fig. 3A) (*20*). Visual imagery of the crevasse sidewalls followed this temperature change, as white meteoric ice in the above freezing water column transitioned in color and texture to green masses of crystalline marine ice in the upper reaches of the crevasse where waters were supercooled (Fig. 4). Interleaving in the salinity profiles resulted from a weakly unstable intracrevasse water column in places, where fluid of somewhat higher salinity and therefore density overlaid fresher and lighter fluid (Fig. 3B). These density imbalances led to vertically propagating instabilities, which are evidenced by stratification frequencies (*N*^2^ = −*g*/ρ_0_ δρ/δ*z*) less than 0 s^−2^ (Fig. 3C). On the eastern sidewall, isolated instabilities (−4 × 10^−5^ s^−2^ *> N*^2^ ≤ −1 × 10^−5^ s^−2^) occurred around 485- and 492-m depth. The shallower instabilities were sampled toward the lower limit of the observed downwelling plume (Fig. 2C) where the otherwise strong sinking velocities abated (fig. S3H). Supercooled conditions (Fig. 3A) and visual evidence of marine ice (Fig. 4) located above these observations suggest that the downwelling plume resulted from marine ice formation releasing heat and salt to densify the water. This is consistent with these data fitting the GMW mixing line that relates to ice melting and freezing (fig. S4; Materials and Methods). The instabilities aligned with reduced salinities that could relate to GMW accumulated from melting driven by the plume that pooled at this depth as sinking weakened (Fig. 3D). We attribute the relatively large variations in GMW concentration along the eastern half of the crevasse to fluctuating sampling distances from the sidewall only partially resolving the downwelling plume and complex hydrographic signals from both melting and freezing.

On the western sidewall, stratification frequencies ranged from weakly unstable to weakly stable (−1 × 10^−5^ s^−2^ *> N*^2^ ≤ 2 × 10^−5^ s^−2^) over the 490- to 508-m-depth range of the throughflow jet, which exhibited broad upwelling (Fig. 2). The approximately neutral stratification over the throughflow jet’s depth range suggests that it efficiently mixed this region of water column. These *N*^2^ measurements were located on average closer to the western crevasse sidewall than the velocity data, which further suggests that well-mixed conditions persisted toward the ice-ocean interface. Hence, we do not expect substantially increased vertical rising velocities near the western crevasse wall. GMW concentrations reached a maximum of 4.05 ml liter^−1^ in the jet (Fig. 3D) and changed little along the western half of the crevasse’s vertical extent, consistent with low *N*^2^ magnitudes or well-mixed conditions.

### Interactions between ice surface morphology and ocean properties

Morphological features present in the ice surface displayed multiple distinctive patterns that varied with depth in the crevasse relative to the supercooling horizon and ocean circulation (Fig. 4).

### Scallops

Hexagonal scallop formations occupied the above-freezing (melting) section of the western and eastern crevasse walls beneath 476- and 494-m depth, respectively (Fig. 4). Scallops in an ice surface frictionally disrupt the proximal ocean circulation to alter the boundary layer velocity profile, increasing form drag and turbulent kinetic energy (*12*). This creates a persistent eddy feature within each scallop, which drives a self-reinforcing fine-scale melt variation that dictates its orientation, wavelengths, and incision depth (*12*). More research is required to explicitly determine whether scallops increase the total melt rate of a region of ice.

Scallops exhibited quasi-symmetrical axes along the eastern sidewall, with axes oriented in the horizontal and vertical directions of wavelength (λ) ranging from 45 to 78 cm and incision depth (*d*) between 13 and 22 cm (table S1). Symmetrical scallops also occurred on the western sidewall keel at 508-m depth, with λ = 48 to 63 cm and *d* = 10 to 13 cm. Scallops along the western sidewall above this depth (between 482 and 506 m) were instead asymmetric, with an elongated vertical axis of λ = 88 to 157 cm and a horizontal axis of λ = 43 to 78 cm. These asymmetric scallops also exhibited greater incision depths, ranging from 15 to 32 cm.

Along the western wall from 482- to 506-m depth, the scallops’ shorter horizontal axis was oriented north-south (*v*), with crests perpendicular to the throughflow jet’s horizontal southward flow (Figs. 2A and 4 and fig. S2, A and B). We interpret the scallops’ horizontal axis orientation and wavelength to reflect the turbulent eddy size generated by velocity shear of the dominant southward horizontal flow with proximity to the ice. The scallops’ longer vertical axis exhibited crests perpendicular to the adjacent upwelling circulation (Fig. 2C and fig. S2D), which we attribute to turbulence generated by this flow. We interpret that the longer vertical wavelengths result from buoyancy released by melting enhancing near-ice turbulence to drive additional fine-scale melt (*13*), thus broadening and deepening the feature. We do not attribute these vertically elongated and deepened scallops to double diffusion, which has been proposed as a formation mechanism for scallops in much warmer and stratified ocean conditions (*14*). Our observations of a cold, well-mixed water column with background speeds of 6 cm s^−1^ instead suggests shear-driven turbulence as a dominant mechanism for driving fine-scale melt.

The contrasting symmetry and shallower incision depths of scallops along the keel of the western sidewall at 508-m depth suggest weaker near-ice upwelling than observed above in the crevasse. Additionally, the orientation of these scallops is less clear, which we attribute to a greater exposure of the crevasse keel to east-west (*u*) tidally driven currents in the underlying ocean cavity (fig. S1). Scallops along the eastern sidewall followed a similar transition from a more random orientation along the crevasse to keel at 506-m depth to a generally more organized north-south horizontal axis pattern upward in the crevasse to 494-m depth (Fig. 4). These features did not exhibit an elongated vertical axis and had shallower incision depths than the elongated western scallops, which is consistent with our observations of weak vertical ocean currents at these depths (Fig. 2C and fig. S2H). Horizontal currents on the eastern half of the crevasse at these depths were variable with a weakly dominant northward flow (fig. S2, E to G) aligned with the scallops’ horizontal axis.

### Runnels

Runnels extended over a 26-m section of the eastern wall from 470- to 506-m depth, while only occurring over 1 m of the western wall from 475- to 476-m depth (Fig. 4). The orientation and geometry of these runnels are consistent with observations of morphological features melted by either positive or negative localized vertical buoyant fluid motion along a vertical ice face (*22*, *58*).

The runnels along the eastern sidewall initiated as 60- to 120-cm-wide and 30-cm-deep features carved into the marine ice above the supercooling horizon (within sensor uncertainty), located at 471-m depth at the time of sampling. They deepened and widened below this depth, etching into the meteoric ice in the above-freezing portion of the crevasse to a maximum of *d* = 210 cm at 494-m depth, with a respective λ = 242 cm. Below this the features widened but did not become noticeably more incised, reaching a maximum observed λ = 526 cm and *d* = 212 cm at 503-m depth. Runnels were also observed along a 1-m section of the western sidewall that straddled the measured supercooling horizon depth of 476 m. Similar to the eastern sidewall, the runnels deepened and widened as they transitioned from marine ice (λ = 81 to 103 cm, *d* = 82 to 85 cm) to meteoric ice (λ = 143 cm, *d* = 148 cm).

Our observations of a vigorous sinking plume adjacent to the eastern runnels and their progressively deeper incision into the ice with depth in the crevasse agree with a localized melting mechanism. However, the presence of scallops superimposed on the eastern wall runnels below 494-m depth and negligible observed vertical ocean currents indicate that horizontal circulation dominated from this depth to the keel. This is additionally supported by negligible further incision of the runnels despite a twofold widening between 494-m depth and the crevasse keel. We also interpret that the western runnels’ small vertical range results from the primarily horizontal throughflow jet dominating melting along most of the above-freezing portion of this crevasse sidewall.

### Marine ice

Small reflective facies of marine ice 8 to 19 cm across filled in the runnels above the supercooling horizon (Fig. 4), reducing their size with proximity to the crevasse roof until they became indistinguishable. Note that these reflective facies are one to two orders of magnitude larger than the typical size of observed frazil crystals, which are *<*0.10 cm (*44*–*46*). We interpret that these facies are an aggregation of frazil crystals under the influence of direct freezing to the crevasse sidewall. Supercooling reached an observed maximum at the crevasse roof where 72- to 140-cm-wide and 183-cm-long quasi-columnar features extended downward that appeared visually more solid than the underlying marine ice (Fig. 4). This suggests that the accreted ice consolidates over time, which is consistent with cores taken in ice shelves that contain a marine ice layer (*47*).

### Frictional drag exerted by ice morphology on the ocean

The immobile crevasse sidewalls exerted drag on ocean flow through the crevasse, which induced velocity shear as it transitioned from free-stream to no flow across the boundary layer. This phenomenon can be represented by the friction velocity (*u*_∗_), which is the square root of the kinematic shear stress exerted by a surface on a flow (Materials and Methods). At the time of sampling, the throughflow jet adjacent to the western wall exhibited a dominant southward horizontal and secondary vertical circulation pattern from 487-m depth to below the crevasse keel (Fig. 2 and fig. S2). The scallops along the western sidewall over this depth range exerted an average *u*_∗_ = 0.32 cm s^−1^ on the ocean (table S1). Relating *u*_∗_ to the scallops’ horizontal λ produced a dimensionless drag coefficient (*C*_D_) between 2.70 × 10^−3^ and 9.20 × 10^−3^, with a mean of 5.90 × 10^−3^ (Materials and Methods). The point of no flow occurred at a roughness length (*z*_0_) of 3.53 cm from the ice surface or $\frac{1}{9.06}$ to $\frac{1}{2.83}$ (mean = $\frac{1}{6.29}$) of the scallops’ *d*.

Scallops along the eastern sidewall from 483- to 506-m depth modified the weaker and less-organized horizontal flow on this half of the crevasse to exert an average *u*_∗_ = 0.26 cm s^−1^. The associated *C*_D_ was slightly larger than the western wall, with a larger range from 4.90 × 10^−3^ to 14.60 × 10^−3^, (mean = 7.70 × 10^−3^). The *z*_0_ was smaller at 2.21 cm with a narrower range of $\frac{1}{9.95}$ to $\frac{1}{5.88}$ (mean = $\frac{1}{7.99}$) of the scallops’ *d*. Despite a much smaller sample number (*N* = 70) used to derive these parameters along the eastern sidewall than the western sidewall (*N* = 839), the scallops throughout the crevasse exerted a similar frictional drag on the adjacent oceanic flow.

The downwelling plume adjoined vertically oriented runnels along the eastern sidewall over an observed depth range of 471 to 483 m. Implementing the same analysis as above yielded *u*_∗_ = 0.21 cm s^−1^ and *z*_0_ = 4.16 cm. This translated to 1 × 10^−3^ ≥ *C*_D_ ≤ 5.50 × 10^−3^ (mean = 2.30 × 10^−3^) based on the runnels’ horizontal λ and $\frac{1}{24}$ to $\frac{1}{2.40}$ (mean = $\frac{1}{8.90}$) of the runnels’ *d*. Note that while the analysis yielded a satisfactory fit (table S1), the lack of data located further away from the wall and the localized nature of runnel melt features cause uncertainty regarding the shape of the boundary layer velocity profile. We suspect that *d* may relate to *u*_∗_ and *C*_D_ in these vertically oriented features more appropriately than λ. Nonetheless, the quantification of frictional forces exerted by various ice morphology on the ocean reveals that they induce shear to increase turbulent kinetic energy, which potentially enhances melting throughout the crevasse.

### Variable melting and freezing rates along crevasse sidewalls

Considering phase changes occurring both directly at the sidewall ice-ocean interface ($\dot{m}$) and at the boundary of frazil crystals ($\dot{f}$) suspended in the water column, we estimated ice melt ($\dot{m}+\dot{f}$ > 0) and freeze rates ($\dot{m}+\dot{f}$ < 0) in the crevasse (Fig. 3F; Materials and Methods). To do this, we used observed ocean temperatures, salinities, and current speeds, except where no velocity data existed in the western half of the crevasse above 486-m depth and the eastern half above 471-m depth. We simulated vertical ocean currents over the unsampled section of the crevasse (Fig. 3E) with a one-dimensional plume circulation model (*59*) and combined observed hydrographic conditions with modeled speeds to estimate melting and freezing (Materials and Methods).

The transition from above to below freezing ocean temperatures paired with highly variable current speeds (Fig. 3E) affected the rate of ice melt and freeze in the crevasse (Fig. 3F). Maximum estimated melt rates ($\dot{m}$ > 0) up to 0.21 ± 0.06 m year^−1^ occurred along the lower 10 m of the western sidewall (below 498-m depth) where shear induced by the throughflow jet efficiently mixed the sparse ocean heat into the ice face. Melt rates along the eastern wall were lower at a maximum around 0.17 ± 0.04 m year^−1^, due to reduced current speeds on this side of the crevasse. Melting decreased with height in the crevasse to near zero at 486-m depth, as current speeds reached a minimum and pressure release caused ocean thermal driving to approach 0°C. The elevated melt rates along the lower western crevasse sidewall relative to the eastern sidewall are consistent with its shallower slope. This suggests that the throughflow jet is a persistent feature that enhances localized melting and erodes this section of the crevasse to produce its shallower slope.

Modeled upwelling flow speeds changed little on the western side between 486 m depth and the crevasse roof. Simulated freeze rates steadily rose from the supercooling horizon at 475-m depth to a maximum at the crevasse roof of −0.18 ± 0.08 m year^−1^, with frazil crystal formation ($\dot{f}$ < 0) contributing 63 to 71% and direct freezing ($\dot{m}$ < 0) contributing 29 to 37% of the total. On the eastern half of the crevasse, no velocity data existed above 471-m depth; however, the observed downwelling plume between 471 and 485-m depth concentrated on the eastern sidewall exhibited heightened flow speeds relative to lower in the crevasse. Modeled freeze rates on the eastern side increased with depth away from the crevasse roof as sinking accelerated and reached a maximum of −0.15 ± 0.07 m year^−1^ just above the supercooling horizon at 470-m depth. The simulated freeze rates were dominated by frazil crystal formation, with $\dot{f}$ contributing 64 to 71% and $\dot{m}$ contributing 29 to 36% of the total rate, respectively. The observed sinking plume extended below the supercooling horizon for about 15- to 485-m depth into temperatures slightly above freezing, and drove weak melting of 0.01 to 0.06 m year^−1^. We interpret that the thinner supercooled layer and dominant sinking motion on the eastern half of the crevasse resulted from the salt and latent heat released through direct freezing on this ice surface and the accumulated frazil ice formation on both sides of the crevasse, which reversed the direction of the western upwelling plume.

The location of runnels in conjunction with the observed vigorous sinking velocities suggests that latent heat and salt rejection from marine ice formation was concentrated along the eastern crevasse sidewall, which argues for a greater contribution from direct freezing to the total freeze rate. While our modeled values of $\dot{m}$ and $\dot{f}$ disagree with this interpretation, we anticipate that these simulations underestimate the intensity of $\dot{m}$ because they ignore horizontal components of the velocity, which approximately double to triple the observed current speed magnitude of the downwelling plume below the supercooling horizon (Fig. 3E). Doubling or tripling the simulated speed along the eastern crevasse wall above the supercooling horizon results in a maximum direct freezing rate of $\dot{m}$ = −0.14 ± 0.08 m year^−1^ or $\dot{m}$ = −0.19 ± 0.12 m year^−1^, respectively, which then contributes 60 to 70% or 68 to 78% of the total freezing. Nevertheless, simulated vertical currents allow for melt and freeze rate estimates in the upper portion lacking velocity data, which provide a useful means of understanding overturning within the crevasse.

### Heat and salt transfer to the ice-ocean interface

Transfer velocities ($\gamma_{T}^{b},\gamma_{S}^{b}$) describe the efficiency of ocean-driven heat and salt movement toward the ice in the melting and freezing rate parameterization, considering both turbulent and molecular processes (Materials and Methods). These velocities are commonly replaced with dimensionless heat and salt transfer coefficients (${\text{Γ}}_{T}^{b},{\text{Γ}}_{S}^{b}$) or thermal and haline Stanton numbers ($\sqrt{C_{D}}{\text{Γ}}_{T}^{b},\sqrt{C_{D}}{\text{Γ}}_{S}^{b})$ in parameterizations of melting and freezing rates when insufficient data coverage prevents direct calculation of $\gamma_{T}^{b},\gamma_{S}^{b}$ (and *C*_D_ in the case of Stanton numbers). We convert our transfer velocities to average values of these parameters (Materials and Methods) so that they may be considered in future studies that are unable to directly calculate $\gamma_{T}^{b},\gamma_{S}^{b}$ and *C*_D_. Table 1 summarizes these parameters for the portion of the crevasse dominated by a horizontal circulation pattern and scalloped ice morphology, and the section along the eastern crevasse sidewall characterized by downwelling and ice runnels. The parameter values lie toward the upper end of the range of published values from other observations beneath ice shelves (*19*, *60*–*62*). This is consistent with very low levels of ocean stratification in the crevasse allowing for efficient heat and salt movement toward the ice, typical of cold ocean cavity ice shelves.

**Table 1.** Parameterizations of heat and salt transfer toward the ice. The summarized values of commonly used heat and salt transfer coefficients for scallops and runnels from this study are compared to other observationally based published studies beneath ice shelves.

	${\text{Γ}}_{T}^{b}$	${\text{Γ}}_{S}^{b}$	${\text{Γ}}_{\left\{TS\right\}}^{b}$	$\frac{{\text{Γ}}_{T}^{b}}{{\text{Γ}}_{S}^{b}}$	*C*_D_	$\sqrt{C_{D}}{\text{Γ}}_{T}^{b}$	$\sqrt{C_{D}}{\text{Γ}}_{S}^{b}$	$\sqrt{C_{D}}{\text{Γ}}_{\left\{TS\right\}}^{b}$
This study								
Scallops	12 × 10^−3^	4.40 × 10^−4^	–	27.27	6.80 × 10^−3^	9.85 × 10^−4^	3.70 × 10^−5^	–
Runnels	9 × 10^−3^	4.25 × 10^−4^	–	21.18	2.30 × 10^−3^	4.61 × 10^−4^	2.26 × 10^−5^	–
Other studies								
(*60*)	11 × 10^−3^	3.10 × 10^−4^	6 × 10^−3^	35.48	9.7 × 10^−3^	11 × 10^−4^	3.10 × 10^−5^	5.90 × 10^−4^
(*61*)	23.5 × 10^−3^	6.70 × 10^−4^	–	35	2.20 × 10^−3^	11 × 10^−4^	–	–
(*62*)	–	–	–	25	–	2.18 × 10^−4^	8.70 × 10^−6^	–
(*19*)	–	–	–	–	–	–	–	0.87 × 10^−4^

### Spring-neap tidal modulation of conditions in the crevasse

The visual onset of marine ice on the eastern sidewall coincided with the depth that neighboring ocean temperatures transitioned to supercooled (*20*). However, marine ice became visible on the western sidewall 1 m below the supercooling horizon (*20*), suggesting that at the time of sampling the ocean was melting this marine ice (within bin-averaged sensor uncertainty). These crevasse observations occurred around slack tide during relatively low tidal amplitudes when the dominant east-west flow in the underlying ocean cavity abated (fig. S1). The first dive with *Icefin* beneath RIS occurred during higher tidal amplitudes when stronger currents mixed heat upward throughout the ocean cavity (fig. S3, A to C). A portion of this dive extending upward to 492-m depth in the crevasse located around 4100 m from the GL (Fig. 1C) confirmed that this upward heat flux extended into the crevasse. Differences in Θ between vertical profiles from this crevasse and the main one studied in this effort (fig. S3D) indicate that crevasse ocean conditions were on average 0.0191°C warmer during spring tide (Materials and Methods). Respective differences in *S*_A_ and σ_Θ_ were negligible at 0.0014 g kg^−1^ and 0.007 kg m^−3^, within the bin-averaged sensor uncertainties (fig. S3, E and F).

This ocean property change would elevate temperatures above freezing throughout the crevasse and prevent any supercooling (Fig. 3A). Assuming the same current speeds, spring tide ocean-driven melt rates approximately double throughout most of the crevasse, with a maximum of 0.53 ± 0.15 m year^−1^ occurring along the lower western sidewall and a somewhat lower maximum of 0.35 ± 0.10 m year^−1^ on the lower eastern sidewall (Fig. 3F). The discrepancy between estimated melting along the sidewalls during spring tide is greater than in neap conditions. This suggests that local melt enhancement along the lower western sidewall could be more pronounced during spring tides, which would further erode this section of the crevasse and reduce its slope.

Predicted spring tide melting persists in the upper portion of the crevasse that was supercooled during neap tide at low rates of 0.03 to 0.12 m year^−1^. The visual imagery of accumulated marine ice along the upper crevasse walls and roof of up to 183 cm thick (column features; Fig. 4) suggests that this layer is not ephemeral, which argues that neap tide freezing dominates spring tide melting (|$\dot{m}$ + $\dot{f}$|_neap_ *>* $\dot{m}$_spring_). The tuned rising and sinking plume model reflects this, with simulated total neap tide freeze rates increasing from ∼65% greater than spring melt rates near the western supercooling horizon to ∼500% greater near the crevasse roof (see Materials and Methods for details).

## DISCUSSION

**Fig. 5.**
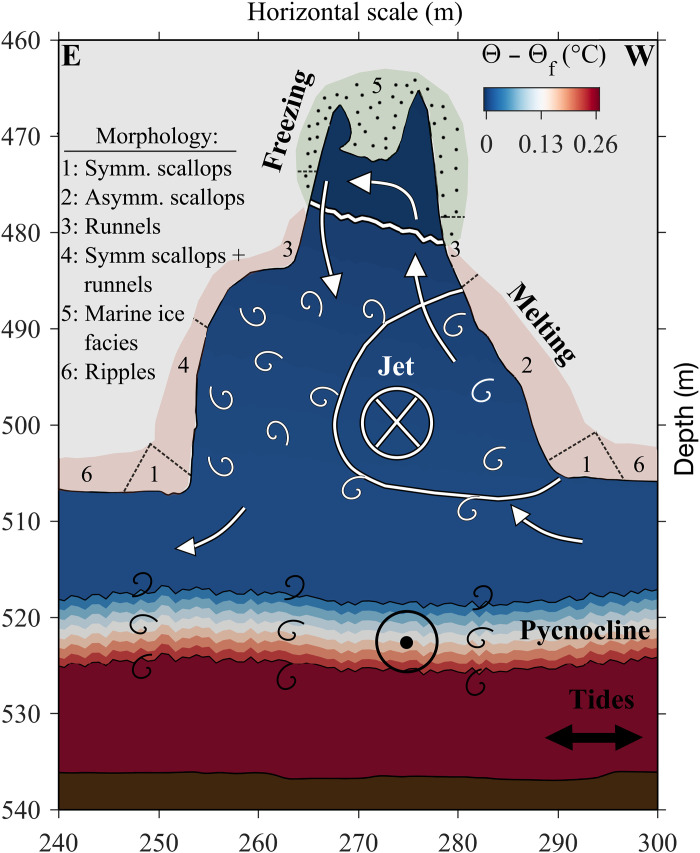
Summary of ocean conditions and ice-ocean interactions within and below the crevasse. Ocean thermal driving decreases with height in the water column and falls below 0°C in the upper portion of the crevasse, leading to a transition from melting to freezing. A jet flowing through the crevasse dominates circulation within the feature and increases melting along the lower western crevasse sidewall. Velocity shear between this jet and the sidewall, as well as the adjacent ocean drives mixing. An overturning circulation overlies this jet, which results from buoyancy dynamics related to ice melting and freezing. The ice morphology within the crevasse varies relative to the ocean circulation pattern and thermal driving. Tides dominate the circulation in the underlying ocean cavity, driving flow perpendicular to the crevasse. The tidal currents mix heat and salt upwards through the pycnocline into the crevasse at variable rates that depend on the tidal phase. Circulation patterns alter across the sharp pycnocline, which could relate to internal waves, small-scale mixing within the pycnocline, or baroclinic flow patterns associated with larger-scale density variations. Note that horizontal scale is identical to Fig. 2.

Our observations expose in unprecedented detail the ice morphology, three-dimensional ocean circulation, and thermodynamics within an ice shelf basal crevasse. We identify a dominant jet flowing through the crevasse and an overturning circulation driven by freshwater release from melting on the lower sidewalls and salt rejection from freezing above (Fig. 5). This overturning flow is consistent with one-dimensional (*59*) and two-dimensional (*63*) ocean circulation modeling of a larger rectangular basal ice shelf rift inspired by prior observations (*31*, *32*), revealing that similar dynamics operate at smaller scales in crevasses of different geometries. However, the extent to which water funneled through crevasses was previously unknown.

The dominant horizontal throughflow jet intensifies estimated melting by four times along the lower western sidewall, when compared to rates driven solely by the overturning crevasse circulation. The higher velocities in this jet more efficiently entrain ambient water from the center of the crevasse, which dilutes cold and fresh GMW released during melting and preserves the upwelling plume’s positive thermal driving. This process raises the supercooling horizon along the western half of the crevasse to the depth predicted solely by pressure release, thus largely negating the cooling and freshening from melting. However, we posit that GMW accumulation from the integrated melting driven by the throughflow jet along the crevasse sidewall will lower thermal driving and the supercooling horizon downstream (south at the time of sampling) of our study site, which would in turn influence crevasse geometry.

The portion of the *Icefin* survey conducted outside of the crevasse identified quasi-two-dimensional ripple formations in the ice base with crests oriented orthogonal to the northeast-southwest (perpendicular to GL) tidal currents that dominated circulation to first order in the underlying ocean cavity (Fig. 5 and fig. S1) (*20*). Laboratory experiments conducted with much warmer water (of 0 g kg^−1^ salinity) and faster current speeds that drove melt rates on the order of 100 m year^−1^ showed that ice morphological features can form in under 12 hours (*12*). However, given that the estimated melt rates in our study region are two to the three orders of magnitude lower (Fig. 3F) (*20*), then we expect the features to form over several months to a year and to reflect the dominant flow direction responsible for driving melting. The scallops in the western crevasse sidewall were oriented ~90° out of phase from those occupying the underlying ice base, which supports our interpretation of an enduring jet flowing through the crevasse in the north-south (parallel to GL) direction. We hypothesize that the jet is a buoyancy-driven flow feature that occurs when tidal currents relax in the underlying cavity. The weakened tidal flow allows for GMW produced from melting along the ice base and mixed into the water column to rise into the crevasse and flow parallel to the GL. Because of the limited temporal sampling of our observations, we cannot determine whether this jet reverses direction from southward to northward. Prior work found that scallops have steeper slopes on the upstream side than downstream due to differential melting (*12*). An analysis of the scallops’ horizontal axis sides showed no discernible difference in slope, which suggests that the jet could reverse direction.

The crevasse width is an order of magnitude lower than the internal deformation radius given the observed ocean conditions, and recorded maximum north-south velocity shear across the crevasse exceeds predicted geostrophic velocities by six times (Materials and Methods). This signifies that Earth’s rotation does not alter the flow direction as it feels the friction of the crevasse wall (*57*) and indicates that the orientation of scallops reflects that of the mean flow. Therefore, the orientation of scallops along the western crevasse sidewall supports our interpretation of a persistent jet flowing in through the crevasse in the north-south direction. The imagery of green marine ice above the supercooling horizon in the crevasse also defends the hypothesis that green icebergs around Antarctica originate below cold cavity ice shelves where various mineral and organic debris can be entrained into the ice and cause a color shift (*64*).

While our data suggest that no freezing occurs in the observed crevasse during periods of larger tidal amplitudes, the greater efficiency of marine ice freezing to melting will likely preserve the crevasse’s infilled geometry unless long-term ocean conditions change. In the cold ocean cavity at our study site, there is minimal stratification in the near-ice boundary layer to suppress melt along the quasi-horizontal ice base outside of the crevasse (*20*). Therefore, it is reasonable to suggest that the ice base melts upward over time at rates similar to what we estimate in the lower half of the crevasse until reaching the long-term mean supercooling horizon where marine ice first became visible. Given a mean melt rate of 0.26 m year^−1^ on the ice shelf keel (*20*), the crevasse will melt away and only leave the portion above the supercooled horizon in 127 years. During this time, the crevasse will widen from lateral melt along its sidewalls to an extent balanced by viscous deformation in the ice column (*65*), and we expect the internal ocean circulation to change with the evolving crevasse’s aspect ratio (*63*).

The dominant jet flowing through the crevasse transported a volume of water southward at 5.54 m^3^ s^−1^ through a 110-m^2^ area between 490- and 512-m depth along the western sidewall. This is four to five times the estimated volumetric overturning rate, when the crevasse half-widths are extrapolated from a horizontal distance (west: 15.31 m at 495-m depth, east: 7.94 m at 479-m depth) to a 110-m^2^ area to convert area fluxes (west: 0.15 m^2^ s^−1^, east: −0.11 m^2^ s^−1^) to volume fluxes (west: 1.09 m^3^ s^−1^, east: −1.52 m^3^ s^−1^). Meltwater only comprised ∼0.40% of the jet’s volume transport due to the low GMW concentrations from relatively weak ice shelf melting. Nevertheless, the nontrivial total volume funneled through the crevasse in this jet at the time of sampling argues that the crevasse extends laterally across RIS for a considerable distance.

The other four crevasses mapped in the under-ice survey all exhibited a shallower slope on their lower western sidewall (Fig. 1C), similar to what we observed proximal to the throughflow jet in the crevasse studied in detail (Fig. 2A). This suggests that a comparable circulation feature exists in each of these crevasses, which drives enhanced localized melt and communicates meltwater and ocean properties parallel to the Kamb Ice Stream GL. Assuming that a jet of similar magnitude exists in each of these crevasses, then the total southward volume transport rate through these features would be ∼27.50 m^3^ s^−1^ over a 550-m^2^ area at the time of sampling. The small area that this water funneled through obscures the potential larger influence of this transport. If the volume transport rate through the crevasses is standardized by the flux gate area, then it becomes 28% of the inflow through one of the main pathways that translates the source ocean water mass (HSSW) beneath RIS that feeds our study region (*66*). Such capacity to efficiently communicate ocean properties and meltwater therefore makes the abundance of crevasses important to the larger-scale ocean circulation in the area of RIS seaward of Kamb Ice Stream, which regional ocean models typically predict to be very isolated (*67*, *68*).

Recent observations beneath the Thwaites Eastern Ice Shelf also show water freely flowing through the lower section of a crevasse in the GZ ocean cavity (*24*). Estimated melt rates at Thwaites are approximately 10 times higher along the crevasse sidewalls than those on the underlying flat ice shelf base (*24*), due to the stabilizing effect of high boundary layer stratification under low basal slopes in warm ocean cavities. These additional observations paired with those discussed here reveal that basal crevasses play an important role in circulating meltwater and ocean properties parallel to the GL of Antarctic ice shelves that occupy wide embayments, much like how basal channels oriented perpendicular to GLs export water away (*62*). Such features represent a source of ocean variability in the region most critical for ice shelf integrity and buttressing of seaward ice flux (*26*), and are vulnerable to increased melting from positive anomalies in ocean forcing (*29*). This work demonstrates the profound effect that small-scale ice features have on ocean-driven ice shelf melting and freezing and motivates improved resolution of coupled regional ice sheet–ocean models to accurately simulate current and future Antarctic Ice Sheet contributions to global mean sea level rise.

## MATERIALS AND METHODS

### Data collection

The data presented here are a subset of a ∼1500-m-long spatial survey (Fig. 1, B and C) that was collected beneath RIS with *Icefin*, oriented approximately parallel to the direction of ice flow and discussed in (*20*). This survey comprised three dives that were conducted between 17 and 21 December 2019 as tidal amplitudes receded from spring toward neap conditions (fig. S1). The final portion of dive 3 was dedicated to collecting data in the crevasse outlined with a red box in Fig. 1C; this occurred around slack tide (fig. S1). Some data from dive 1 were collected in the crevasse located around 4750 m from the GL that is compared to the main crevasse dataset. *Icefin* carries a suite of sensors onboard that monitor the hydrographic properties of the water column and the ice and seafloor elevations. It also hosts several video cameras that provide visual imagery of the under-ice environment and a forward-looking sonar that can be used to measure range from a surface and the dimensions of any morphological features present on that surface. Further discussion of *Icefin* vehicle design, deployment, and data processing are also found in (*20*, *24*).

### Navigation solution

Under-ice vehicle navigation was accomplished through dead reckoning with a LinkQuest NavQuest 600 Micro Doppler velocity logger (DVL) onboard *Icefin* that determines three-dimensional velocities based on the Doppler shift of acoustic signals reflecting off a surface. These initial velocities relate to the vehicle’s major, minor, and vertical axes and must be combined with a heading provided by *Icefin*’s inertial measurement unit to convert motion to Earth’s reference frame. An initial latitude and longitude taken on the ice surface provides the start point of each dive, and the integrated under-ice velocity and heading data produce the resultant vehicle track. Because this form of navigation depends on integrated velocity and heading data, the location uncertainty also integrates over the track, with the number of dropped acoustic pings from the DVL increasing the uncertainty. Highly variable vehicle dynamics while navigating inside of the crevasse led to many dropped pings from the DVL and a location uncertainty on the order of tens of meters upon dive completion. Therefore, the ice and seafloor elevations in Fig. 2 come from the main outbound leg of dive 3 (fig. S1) when location uncertainty was low, and we corrected the vehicle track inside of the crevasse with the ranges from crevasse walls collected with the forward-looking sonar (Fig. 2). We averaged the vehicle data and locations along each crevasse wall into 1-m bins to further reduce location uncertainty. Portions of the vehicle track above the ice base reveal discrepancies between the ice elevation recorded on the outbound leg of the dive and the crevasse geometry that *Icefin* interrogated upon return. We attribute these discrepancies to the rough crevasse roof morphology that comprised meter-long columnar marine ice features (Fig. 4) and a prevailing southward flow beneath the crevasse (Fig. 2A and fig. S1) that transported *Icefin* away from where it sampled ice elevations on the outbound leg of the dive. Nonetheless, the ability to scale ranges from the crevasse walls with the forward-looking sonar and the general agreement of ice and vehicle track elevation solidifies the relative location of *Icefin* inside the crevasse.

### Postprocessing of in situ hydrographic data

We follow the processing reported in (*20*, *24*), with the exception of an additional step 7 (see below). The in situ hydrographic data presented here come from two sensors on board *Icefin*: a Neil Brown Ocean Sensors conductivity-temperature (*CT*) sensor and a Valeport ultraP pressure (*P*) sensor. The manufacturer-stated accuracies are *C*: ± 0.010 mS cm^−1^, *T*: ± 0.005°C, and *P*: 0.100 dbar. The *C*, *T*, and *P* accuracies translate to uncertainties in *S*_A_ of ±0.008 g kg^−1^ and Θ of ±0.018°C. All sensors were factory calibrated before entering the field in 2019, and the CT sensor was field calibrated through a cross comparison of vertical profile data from beneath the borehole at RIS with data obtained independently with an RBR Concerto3 CTD profiler. This resulted in offsets/root mean square residuals of *C*: 0.249 mS cm^−1^/0.003 mS cm^−1^ and *T*: 0.018°C/0.002°C. The fact that these residuals are lower than the manufacturer-stated accuracies lends confidence to the quality of data after application. The CT sensor recorded at a frequency of 5 Hz and the *P* sensor recorded at 1 Hz. Pressure measurements were interpolated to match the 5-Hz CT data to derive hydrographic variables.

The CTD data were separated into two tracks, one along either wall of the crevasse (Fig. 2) and postprocessed with the following steps:

1) Remove background atmospheric pressure reading;

2) Remove outliers ±2 standard deviations (SDs) from the mean for *C* and *T* (excluding borehole data);

3) Apply three-point weighted mean filter to *C*, *T*, and *P*;

4) Align *C* and *T* measurements with time lag (upon investigation, a 0-s lag produced the best results);

5) Remove *C* and *T* data when *Icefin* moved at speeds less than 5 cm s^−1^;

6) Derive hydrographic variables such as Θ, *S*_A_, density (ρ), etc., using the Thermodynamic Equation of Seawater 2010 (*69*); and

7) Average hydrographic variables into 1-m bins with a ±1 SD range.

After postprocessing, a mean of 77 and 73 samples were averaged into each 1-m bin of the associated hydrographic variable for the west and east crevasse profiles, respectively. The average ±1 SD range for each 1-m bin of *S*_A_ and Θ were 0.002 g kg^−1^ and 0.002°C for the west profile and 0.002 g kg^−1^ and 0.003°C for the east profile. These ranges are well below the initial uncertainties derived from manufacturer-stated *C*, *T*, and *P* accuracies, which supports our analysis and interpretation of small-scale variability in these properties within the crevasse.

### Postprocessing of ocean current velocities

We follow the processing reported in (*20*, *24*); however, those papers did not present velocities in the *z* direction discussed here. Three-dimensional ocean current velocities come from the DVL on board *Icefin*, which doubles as an acoustic Doppler current profiler (ADCP). As the DVL retrieves vehicle motion from an immobile surface, the ADCP function monitors water column velocities in 2-m bins at a variable start distance from the vehicle. The minimum nominal altitude from a surface for the ADCP to operate is 10 m, and gradients in vehicle pitch, roll, heading, and velocity control the distance of the first bin from the vehicle and the sampling frequency. Here, we subsample ADCP velocities to a fixed rate of 1 Hz; they can reach a maximum of 5 Hz. Manufacturer-stated accuracies for our ADCP are 1% of the DVL-recorded vehicle velocity in that direction. *Icefin* typically travels at ≤50 cm s^−1^ in the *x* direction, making uncertainty in ADCP × velocities 0.50 cm s^−1^. Vehicle motions in *y* and *z* are typically substantially slower, so the associated ADCP *y* and *z* uncertainties are also lower.

The ADCP *x*, *y*, and *z* velocity components were separated into two groups, one on either side of the crevasse (Fig. 2) and postprocessed with the following steps:

1) Remove measurements if bin depth is below seafloor depth;

2) Recalculate bin depth and horizontal location considering vehicle pitch and roll;

3) Remove measurements if exactly 0 m s^−1^ or unadjusted (before differencing vehicle velocity) value is 32,767 m s^−1^ (the default no-data value);

4) Remove measurements if absolute value of vehicle pitch or roll is greater than 30°;

5) Recalculate measurements considering vehicle pitch and roll (excluding *x* and *y* velocities);

6) Convert measurements from vehicle reference frame to geographic reference frame (excluding *z* velocities);

7) Apply 30-s rolling mean filter;

8) Filter measurements for gradients greater than 1 SD from the mean in vehicle speed, pitch, roll, and individual bin velocity; and

9) Collate bins into 1-m vertical bins and then remove measurements if they exceed 1 SD of the mean for that 1-m depth range.

After postprocessing, the ADCP *x*, *y*, and *z* velocity components reflect ocean currents in the eastward (*u*), northward (*v*), and vertical (*w*) directions.

### Postprocessing of ice and seafloor elevations

We follow the processing reported in (*20*, *24*). Ice base and seafloor elevations presented here come from an Impact Subsea ISA500 altimeter and the abovementioned DVL. During the crevasse dive, the altimeter was oriented upward and the DVL was oriented downward. The DVL manufacturer states a range-finding accuracy of ±10 cm. This requires an accurate input of local water column sound speed, which we set appropriately before deploying *Icefin*. The altimeter manufacturer states an accuracy of up to 1 mm. The DVL takes into account pitch, roll, and heading when producing ranges, and we apply these corrections to the altimeter in postprocessing.

The elevation data from each of these sensors were postprocessed in the following manner:

1) Remove data when vehicle pitch or roll exceeds 30°;

2) Remove data when the gradient in vehicle pitch, roll, or heading exceeds 1 SD of the respective mean;

3) Despike by removing data that exceed 2 SDs of the mean of the gradient in elevation of the surface;

4) Investigate elevation profiles and manually remove outliers; and

5) Apply a 3-point weighted mean filter to ice elevations and an 11-point weighted mean filter to seafloor elevations.

After postprocessing, 90% of the ice elevation measurements were at a horizontal resolution of 63 ± 59 cm (mean ± 1 SD), with a minimum and maximum spacing of 0.06 and 627 cm, respectively. Eighty-six percent of postprocessed seafloor data were at a horizontal resolution of 44 ± 23 cm (mean ± 1 SD), with a minimum and maximum spacing of 18 and 169 cm, respectively.

### Calculating Θ, *S*_A_, and σ_Θ_ differences between dive 1 and dive 3

During dive 1, a small subset of data were collected up to 492-m depth in the crevasse located around 4750 m from the GL (Fig. 1C). These data were acquired closer to spring tide when faster ocean currents (fig. S1, B and C) mixed heat and salt upward into the crevasse (fig. S3) (*20*). We postprocessed the crevasse data from dive 1 following the steps listed above then computed Θ, *S*_A_, and σ_Θ_ differences at each depth bin from 492 to 535 m between dive 1 and dive 3 (fig. S3). Note that we averaged dive 3 east and west profiles into a mean “central” crevasse profile in this exercise, because the dive 1 data were located toward the crevasse’s center. Dive 1 and 3 profiles exhibited very little change in Θ, *S*_A_, and σ_Θ_ with depth between the upper layer and in the crevasse, large variability across the pycnocline, and contrasting behaviors in the lower layer (fig. S3, A to C). The differences between profiles reflected these patterns (fig. S3, D to F), which led us to select the upper and crevasse layers for our offsets. We decided this because variability was most consistent between profiles in these layers, and the target of this effort is to investigate ice-ocean thermodynamics in an ice shelf basal crevasse. The Θ, *S*_A_, and σ_Θ_ differences in the upper and crevasse layers ranged from 0.0091° to 0.0293°C, from −0.0041 to 0.0052 g kg^−1^, and from −0.0037 to 0.0038 kg m^−3^, respectively. We selected the mean difference of Θ = 0.0191°C, *S*_A_ = 0.0014 g kg^−1^, and σ_Θ_ = 0.007 kg m^−3^, and applied each to the dive 3 profiles as constant offsets to predict hydrographic conditions in the crevasse during spring tide. Note that the *S_A_* and σ_Θ_ differences are within the uncertainty range of the bin-averaged data and thus not significant.

### Water mass partition

We estimate the concentrations of water masses in the ocean cavity beneath RIS with a three-point endmember partition (*39*) that is identical to the approach of (*20*). This method assumes that Θ and *S*_A_ changes reflect the turbulent mixture between three water masses:

1) HSSW formed in Ross Sea polynyas during winter (*36*, *37*): Θ = −1.90°C, *S*_A_ = 34.93 g kg^−1^;

2) Fresh GMW produced by ocean-driven ice shelf melt (*39*): Θ = 92.50°C, *S*_A_ = 0 g kg^−1^; and

3) Subglacial freshwater (SGW) produced by geothermal and frictional heating of the ice upstream of the GL (*70*) that then discharges across the GL into the ocean: Θ = −0.39°C, *S*_A_ = 0 g kg^−1^.

The HSSW hydrographic properties reflect those present at the RIS front in McMurdo Sound, which we sampled prior to the RIS GZ fieldwork. Admixture of GMW from ocean-driven melt by HSSW forms a straight line between these two water masses with a slope of around −2.40°C (g kg^−1^)^−1^ determined by the following equation (*39*)

$$\text{Θ}(S_{A})={\text{Θ}}_{\mathrm{HSSW}}+\frac{L_{f}}{c_{p}}\left(1-\frac{S_{A_{\mathrm{HSSW}}}}{S_{A}}\right)$$ (1)

Θ_HSSW_ and *S*_A_HSSW__ reflect the hydrographic properties of HSSW, Θ and *S*_A_ are the evolving properties along the GMW mixing line, *L_f_* = 3.34 × 10^5^ J kg^−1^ is the latent heat of fusion, and *c_p_* = 3986 J kg^−1^°C^−1^ is the specific heat capacity of cold seawater at a salinity of *S*_A_HSSW__.

The production of SGW does not remove the latent heat from the ocean to convert solid ice to liquid water, so its Θ represents the freezing point of freshwater at the estimated GL pressure of 543.50 db. Therefore, the input of SGW into the ocean results in a small effect on Θ relative to *S*_A_ change, which distinguishes it from GMW input. Upon investigation of the Θ – *S*_A_ diagram, all hydrographic data fit the HSSW-GMW mixing line within the uncertainty of *Icefin*’s CT sensor. The water mass partition reflected this relationship, with no apparent SGW in the water column during sampling. Note that we multiply water mass fractions by 1000 to present them as milliliters per liter throughout the text. The uncertainty in *Icefin*’s CT sensor results in a GMW uncertainty of 0.200 ml liter^−1^. However, after bin-averaging, the average ± 1 SD range for each 1-m bin of GMW is substantially lower at 0.019 and 0.027 ml liter^−1^ for the west and east profile, respectively.

### Influence of ice morphology on boundary layer drag

We combine ADCP velocities with scaled ice morphologies obtained with *Icefin*’s video cameras and forward-looking sonar to investigate the friction exerted by the crevasse walls on ocean flow. We first partition ocean velocity data based on which half of the crevasse they were located in and calculate their horizontal distance from the closest ice surface. We then identify distinct sections along each crevasse wall based on ocean flow and ice morphology characteristics and quantify the frictional force exerted by the ice on the ocean with the logarithmic law of the wall. This relationship assumes that outside of a small sublayer there exists a fully turbulent boundary layer where current speed increases logarithmically away from an immobile surface until reaching free-stream flow, in the absence of rotational effects (*57*). We choose to neglect rotation here because the crevasse width is an order of magnitude lower than the internal deformation radius (shown below with Eq. 31), which determines the horizontal length scale at which depth-dependent rotational effects become important. This is further supported by measured velocity shear exceeding predicted geostrophic velocities by six times (shown below with Eq. 32). The logarithmic law of the wall is as follows

$$U(x)=\frac{u_{*}}{\kappa }\ln(x)-\frac{u_{*}}{\kappa }\ln(z_{0})=\mathrm{aln}(x)+b$$ (2)

where *U*(*x*) denotes current speeds (calculated as *U* = $\sqrt{u^{2}+v^{2}+w^{2}}$) at various horizontal distances from the ice surface. This approach typically considers vertical distance from a surface; however, here we analyze the flow based on horizontal distance from the crevasse walls. The friction velocity *u*_∗_ is the square root of the kinematic shear stress exerted by the surface on the flow, κ = 0.41 is von Karman’s constant, and *z*_0_ represents the roughness length scale associated with no flow. Note that we only consider *U* ≥ 3 cm s^−1^ in this analysis, as prior work has shown this speed to be the lower bound for the transition from buoyancy-controlled to shear-controlled turbulent mixing along a sloping ice wall (*71*). Following the approach of (*72*), we solve for *u*_∗_ and *z*_0_ by finding the slope (*a*) and *y* intercept (*b*) of the boundary layer logarithmic profile along sections of each crevasse wall with distinct circulation patterns. We express the respective *z*_0_ value as a fraction of the morphological incision depth *d* of features along the crevasse wall (table S1).

After obtaining a friction velocity for each section, we relate it to the mean current speed $\bar{U}$ (only considering *U* ≥ 3 cm s^−1^) with the quadratic stress formula (*73*)

$$u_{*}^{2}=C_{D}{\bar{U}}^{2}$$ (3)

yielding a mean drag coefficient *C*_D_. We then calculate a range of values for *C*_D_ by combining Eq. 3 with an expression that describes the wavelength λ of morphological features as a function of u_*_ (*74*)

$$\lambda =\frac{1000\nu }{\sqrt{C_{D}}\bar{U}}$$ (4)

where ν = 1.95 × 10^−6^ m^2^ s^−1^ is the kinematic viscosity of seawater. Reordering Eq. 4 produces

$$C_{D}={\left(\frac{1000\nu }{\lambda \bar{U}}\right)}^{2}$$ (5)

which then provides an expression for *C*_D_ that depends on the range of ice morphology wavelengths and the mean current speed along a section of the crevasse wall. Table S1 lists ice morphological scales and their impact on shear-driven mixing for different sections of the crevasse.

### Melt and freeze rate parameterization

#### Direct melting and freezing at the crevasse wall interface

We estimate melt and freeze rates along the crevasse walls with a parameterization that describes heat and salt transfer through the ocean boundary layer toward the ice (*75*). This parameterization considers the divergence of heat ($\text{Δ}Q_{B}^{T}$) and salt ($\text{Δ}Q_{B}^{S}$) fluxes at the ice-ocean interface to be balanced by the latent heat ($Q_{{\mathrm{Latent}}_{B}}^{T}$) expended and freshwater ($Q_{{\mathrm{Fresh}}_{B}}^{S}$) released during melting. In this configuration, melting results from a positive ocean heat and salt flux and freezing from a negative flux, which instead releases latent heat and salt. The boundary layer parameterization is as follows

$$T_{B}=aS_{B}+b+cp_{B}$$ (6)

$$\text{Δ}Q_{B}^{T}=Q_{O_{B}}^{T}-Q_{I_{B}}^{T}=Q_{{\mathrm{Latent}}_{B}}^{T}$$ (7)

$$\text{Δ}Q_{B}^{S}=Q_{O_{B}}^{S}-Q_{I_{B}}^{S}=Q_{{\mathrm{Fresh}}_{B}}^{S}$$ (8)

where Eq. 6 represents the in situ temperature (*T_B_*), practical salinity (*S_B_*), and pressure (*p_B_*) at the ice-ocean interface and Eqs. 7 and 8 describe the conservation of heat and salt, respectively. In this relationship *T_B_* resides at the freezing point dictated by the local salinity and pressure; *a* = −5.73 × 10^−2^°C psu^−1^, *b* = 9.39 × 10^−2^, and *c* = −7.53 × 10^−8^°C Pa^−1^ are considered constants. The heat (salt) flux divergence is the difference between the ocean heat (salt) flux toward the ice ($Q_{O_{B}}^{T},Q_{O_{B}}^{S}$) and the diffusive heat (salt) flux through the ice column ($Q_{I_{B}}^{T},Q_{I_{B}}^{S}$), where typically $Q_{I_{B}}^{S}=0$.

We proceed with the conservation of heat equations in the following form

$$Q_{O_{B}}^{T}=\rho_{w}c_{p}\gamma_{T}^{b}(T-T_{B})$$ (9)

$${Q_{I_{B}}^{T}=\rho_{i}c_{p_{i}}\kappa_{i}^{T}\frac{\delta T_{i}}{\delta z}|}_{B}$$ (10)

$$Q_{{\mathrm{Latent}}_{B}}^{T}=\rho_{i}L_{f}\dot{m}$$ (11)

In Eq. 9, ρ*_w_* and *T* denote the measured ocean density and temperature along the crevasse walls, *c_p_* = 3974 J kg^−1^°C^−1^ is once again the specific heat capacity of cold seawater, and $\gamma_{T}^{b}$ is the transfer velocity that describes the mixing of heat toward the ice (formulation described below).

In Eq. 10, ρ*_i_* = 918, *c_p_i__*= 2009 J kg^−1^°C^−1^, and $\kappa_{i}^{T}=1.14$ × 10^−6^ m^2^ s^−1^ are the density, specific heat capacity, and thermal diffusivity of ice, respectively. We assume that the temperature gradient through the ice shelf (${\frac{\delta T_{i}}{\delta z}|}_{B}$) is linear

$${\frac{\delta T_{i}}{\delta z}|}_{B}=\frac{T_{S}-T_{B}}{H_{i}}$$ (12)

so temperatures decrease from the seawater freezing point *T_B_* at the ice-ocean boundary at each depth in the crevasse to the annual mean ice-atmosphere interfacial surface temperature *T_S_* = −26°C, which was measured at the top of the hot water–drilled borehole. The ice surface at our site resided at 71.50 m above sea level, so *H_i_* varies from 580 m at the crevasse base to 537 m at the crevasse roof.

In Eq. 11, *L_f_* = 3.34 × 10^5^ J kg^−1^ is once again the latent heat of fusion, and $\dot{m}$ represents the melt or freeze rate along the crevasse walls, where $\dot{m}$ *>* 0 for melting and $\dot{m}$ *<* 0 for freezing.

The conservation of salt equations resemble those for conservation of heat. With the assumption that $Q_{I_{B}}^{S}=0$, they are as follows

$$Q_{O_{B}}^{S}=\rho_{w}\gamma_{S}^{b}(S-S_{B})$$ (13)

$$Q_{{\mathrm{Fresh}}_{B}}^{T}=\rho_{i}\dot{m}(S_{B}-S_{i})$$ (14)

In Eq. 13, *S* denotes the measured ocean practical salinity along the crevasse walls, and *S_i_* is the ice salinity that we consider to be 0. The transfer velocity that describes the mixing of salt toward the ice is $\gamma_{S}^{b}$. We describe $\gamma_{T}^{b},\gamma_{S}^{b}$ with the following equation from (*59*, *76*)

$$\gamma_{T}^{b}=\frac{\sqrt{C_{D}}U}{2.12\;\ln\left(\frac{\sqrt{C_{D}}UD}{\nu }\right)+12.5\;{Pr}^{2/3}-9}$$ (15)

$$\gamma_{S}^{b}=\frac{\sqrt{C_{D}}U}{2.12\;\ln\left(\frac{\sqrt{C_{D}}UD}{\nu }\right)+12.5\;{Sc}^{2/3}-9}$$ (16)

In Eqs. 15 and 16, turbulent processes that determine mixing throughout most of the ice-ocean boundary layer are described by terms that include $\sqrt{C_{D}}U$, which following Eq. 3 represents the measured *u*_∗_ at each depth along the crevasse walls. The dimensionless Prandtl number *Pr* = 13.8 in Eq. 15 is the ratio of seawater’s viscosity ν = 1.95 × 10^−6^ m^2^ s^−1^ to thermal diffusivity κ*_T_* = 1.4 × 10^−7^ m^2^ s^−1^ and describes molecular thermal diffusion across the viscous sublayer of the ice-ocean boundary layer. Note that the dimensionless Schmidt number *Sc* = 2432, and the ratio of ν to salt diffusivity κ_S_ = 8.0 × 10^−10^ m^2^ s^−1^ in Eq. 16 describes the much slower salt diffusion across the viscous sublayer. We derive the plume thickness *D* at each depth along the west and east crevasse wall using a vertical rising and sinking plume model (described below). Several nondimensional parameters commonly used in Eqs. 9 and 13 relate to $\gamma_{T}^{b},\gamma_{S}^{b}$ when lower data coverage prevents their direct calculation with Eqs. 15 and 16. The dimensionless heat and salt transfer coefficients (${\text{Γ}}_{T}^{b},{\text{Γ}}_{S}^{b}$) represent $\gamma_{T}^{b},\gamma_{S}^{b}$ divided by $\sqrt{C_{D}}U$ and the dimensionless thermal and haline Stanton numbers are $\sqrt{C_{D}}{\text{Γ}}_{T}^{b},\sqrt{C_{D}}{\text{Γ}}_{S}^{b}$.

The three unknowns in the melt rate parameterization are $\dot{m}$*, T_B_*, and *S_B_*. Equations 6 to 14 can be rearranged to substitute out *T_B_* and *S_B_* and solve for $\dot{m}$ with a quadratic expression at each depth bin in the crevasse. Afterward, *T_B_* and *S_B_* can be calculated.

#### Frazil crystal freezing

We follow a similar method to that described in the above section to estimate freeze rates of frazil ice crystals suspended in the supercooled water column by parameterizing heat and salt transfer at the crystal-ocean interface (*59*). We assume that all frazil crystals suspended in the water column adhere to the crevasse walls and roof before supercooled conditions during neap tide transition to above freezing in spring tide. Therefore, we only consider frazil ice freezing and not melting, where once again freezing results from a negative ocean heat and salt flux. The parameterization is as follows

$$T_{C}^{D}=aS_{C}^{D}+b+c\left(p_{B}-\frac{D\cos\theta }{2}\right)$$ (17)

$$Q_{O_{C}}^{T}=Q_{{\mathrm{Latent}}_{C}}^{T}$$ (18)

$$Q_{O_{C}}^{S}=Q_{{\mathrm{Fresh}}_{C}}^{S}$$ (19)

In Eq. 17, $T_{C}^{D},S_{C}^{D}$ represent the hydrographic properties at the crystal-ocean interface in the frazil-laden plume as it rises along the sloping ice base (angle θ) at each depth (pressure *p_B_*). Equations 18 and 19 describe the conservation of heat and salt, where heat diffusion through the frazil crystal is excluded in Eq. 18.

The conservation of heat and salt is as follows

$$Q_{O_{C}}^{T}=(1-C)\rho_{w}c_{p}\gamma_{T}^{c}(T-T_{C}^{D})D\frac{2C}{r}$$ (20)

$$Q_{{\mathrm{Latent}}_{C}}^{T}=\rho_{i}L_{f}\dot{f}$$ (21)

$$Q_{O_{C}}^{S}=(1-C)\rho_{w}\gamma_{S}^{c}(S-S_{C}^{D})D\frac{2C}{r}$$ (22)

$$Q_{{\mathrm{Fresh}}_{C}}^{S}=\rho_{i}\dot{f}(S_{C}^{D}-S_{i})$$ (23)

In Eqs. 20 and 22, *C* represents the fraction of frazil crystals suspended in the water column at any time (varied from 1 × 10^−9^ to 1 × 10^−5^), and in Eqs. 21 and 23, $\dot{f}$ denotes the frazil melt or freeze rate. The hydrographic properties (*T* and *S*) are identical to the observations used at each depth bin in Eqs. 9 to 14 above. The velocity for heat and salt transfer at the crystal-ocean interface $\gamma_{T}^{c},\gamma_{S}^{c}$ is slightly different from $\gamma_{T}^{b},\gamma_{S}^{b}$, as it is strictly a function of molecular diffusivity

$$\gamma_{T}^{c}=\frac{Nu\kappa_{T}}{\epsilon r}$$ (24)

$$\gamma_{T}^{c}=\frac{Nu\kappa_{S}}{\epsilon r}$$ (25)

In Eq. 19 *Nu* = 1 is the dimensionless Nusselt number, the ratio of convective to conductive heat transfer at a boundary, $\epsilon =\frac{1}{16}$ is the aspect ratio of frazil crystals, and *r* is their radius. We choose the respective mean, minimum, and maximum value of *r* to be 0.60, 0.50, and 0.70 mm, which are within the range of observations (*44*) and what has been used in past modeling studies (*59*, *63*). The value used for ε comes from laboratory observations (*77*) and the range of *C* comes from a past modeling study (*59*).

The three unknowns in the frazil parameterization are $\dot{f},T_{C}^{D}$, and $S_{C}^{D}$, which can be substituted and solved for using a quadratic expression similar to that used in Eqs. 6 to 14.

### Vertical rising and sinking plume model

We integrate our hydrographic observations of *T* and *S* over the full crevasse with a subset of equations from the one-dimensional plume model of (*59*) to estimate *w*, *D*, $\dot{m}$, and $\dot{f}$ where we lack the necessary ocean velocity observations to calculate $\dot{m}$ and $\dot{f}$. Note that we consider the full range of *C*_D_ values from table S1 in our simulated estimates of $\dot{m}$ and $\dot{f}$, as we do not have sufficient model data to empirically derive an average u_*_ over the unsampled region of the crevasse. We first achieve a best fit of the model to observations (described below), then use it to solve for *D* over the full crevasse extent along each sidewall. Values of *D* were initially set to half the width of the crevasse to compute $\dot{m}$ at each depth bin, but then, we recalculated $\dot{m}$ with the updated *D* values. The plume model equations are as follows

$$w_{\mathrm{west}}D=w_{\mathrm{east}}(l-D)$$ (26)

$$\frac{\delta }{\delta z}(Dw)=\dot{e}+\dot{m}$$ (27)

$$\frac{\delta }{\delta z}(DwC)=-\frac{\rho_{0}}{\rho_{i}}\dot{f}$$ (28)

$$\frac{\delta }{\delta z}(Dw^{2})=\frac{\left(\rho_{m}-\rho_{a}\right)}{\rho_{0}}Dg\sin\theta -C_{D}w^{2}$$ (29)

Beginning with Eq. 26, we conserve vertical area flux (m^2^ s^−1^) in the region of the crevasse where we lack data in both the western and eastern halves by setting the upwelling flux *w*_west_*D* equal to the downwelling flux *w*_east_(*l* − *D*), where *l* − *D* is the total width of the crevasse minus the upwelling plume width. We make the assumption that *l* − *D* equals the eastern half-width of the crevasse at our uppermost measurement of *w*_east_ (471-m depth), effectively setting the downwelling plume width. We then find a value of *D* at our uppermost measurement of *w*_west_ (487-m depth) that after running the model produces a best fit to *w*_east_(*l* − *D*) at 471-m depth, with a residual of 0.01 m^2^ s^−1^ or ∼10% of the observation. The model iterates in 1-m bins to be consistent with our sampling resolution, considering the conservation of mass (Eqs. 27 and 28) and the conservation of momentum (Eq. 29). In Eq. 27, $\dot{e}$ denotes the entrainment rate

$$\dot{e}=E_{0}w\sin\theta$$ (30)

which is a function of *w*, the sine of the wall slope (θ_west_ = 83° for depth ≤485 m, 58° for depth >485 m; θ_east_ = 75° for depth ≤485 m, 80° for depth >485 m), and the entrainment constant *E*_0_ = 0.07 (*78*). When computing *ė* along the sampled portion of the crevasse, we use the full current speed magnitude *U*. The plume density ρ*_m_* = ρ*_w_* − *C*(ρ*_i_* − ρ*_w_*) and the ambient fluid density ρ*_a_* is the mean of ρ_*w*_west__ and ρ_*w*_east__ at each depth bin; ρ_0_ = 1027 kg m^−3^ is the reference density.

We run the plume model for both neap and spring tidal phases in the crevasse, then compare the spring $\dot{m}$ rates to the neap $\dot{m}$ + $\dot{f}$ rates. Visual imagery shows a layer of marine ice along the upper crevasse walls and roof of up to 183 cm thick (column features; Fig. 4). Given the simulated spring melt rates of ≤0.12 m year^−1^, we interpret that this marine ice does not melt away during spring tides. Therefore, we consider a range of initial values for *C* (1 × 10^−9^ to 1 × 10^−5^) in our model and select the lowest initial value of *C*, which produces an $\dot{f}$ that when added to $\dot{m}$ during neap conditions results in a total freeze rate greater than the spring melt rate on either sidewall. We find that *C*_initial_ = 1 × 10^−6^ fulfills this requirement for the full range of *C*_D_ values considered (Fig. 3F). Note that when *C*_initial_ = 1 × 10^−5^, we estimate very high values of $\dot{f}$ up to 2 m year^−1^ that are unrealistic given the crevasse’s ∼10-m width near the roof.

### Internal deformation radius

We follow (*57*) and compute the internal deformation radius for a two-layer ocean

$$R_{i}=\frac{1}{f}\sqrt{\frac{g^{\prime }H_{1}H_{2}}{H_{1}+H_{2}}}$$ (31)

where $g^{\prime }=g\frac{\left(\rho_{2}-\rho_{1}\right)}{\rho_{2}}$ is the reduced gravity term and *f* = 2 Ωsinϕ is the Coriolis force. The constants in these terms are gravity *g* = 9.81 m s^−2^ and Earth’s rotation Ω = 7.29 × 10^−5^ s^−1^. We consider a mean latitude from the crevasse of ϕ = −82.78°, an upper layer density of ρ_1_ = 1027.88 kg m^−3^ that represents the mean of the full water column above the pycnocline (thickness *H*_1_ = 53 m), and a lower layer density of ρ_2_ = 1027.95 kg m^−3^ that represents the mean of the water column below the pycnocline (thickness *H*_2_ = 12 m); this produces *R_i_* = 559 m. We choose to consider the full water column above the pycnocline as our upper layer, because the vertical density gradient was very weak over this depth range.

### Geostrophic velocities

We follow (*57*) and compute geostrophic circulation with the thermal wind relation

$$\frac{\delta v_{g}}{\delta z}=\frac{-g}{\rho_{0}f}\frac{\delta \rho }{\delta x}$$ (32)

The calculated geostrophic velocity *v_g_* is directed through the crevasse and is based on an assumed level of no flow at our lowest data point (532-m depth). When the lateral density gradients ($\frac{\delta \rho }{\delta x}$) between the east and west profiles are used in Eq. 32, it produces southward flow through the crevasse of *v_g_* = −1 cm s^−1^. This is one-sixth of the maximum observed southward velocities in the throughflow jet.

### Statistics

All statistics were performed in MATLAB. The above sections provide the details of these statistical analyses, with the underlying mean and SD functions being native to MATLAB.

## Supplementary Materials

### This PDF file includes:

Figs. S1 to S4 Table S1 References
