# Supplementary material for: Direct observations of melting, freezing, and ocean circulation in an ice shelf basal crevasse
Source: Sci Adv. 2023 Oct 27;9(43):eadi7638. doi: 10.1126/sciadv.adi7638 (PMC10610921; doi:10.1126/sciadv.adi7638)
Supplement: Supplementary file 1 — Figs. S1 to S4 Table S1 References [file sciadv.adi7638_sm.pdf]

Science Advances
AAAS

Supplementary Materials for

Direct observations of melting, freezing, and ocean circulation in an ice shelf basal crevasse

Peter Washam *et al.*

Corresponding author: Peter Washam, pwasham@cornell.edu

Sci. Adv. **9**, eadi7638 (2023)
DOI: 10.1126/sciadv.adi7638

This PDF file includes:

Figs. S1 to S4
Table S1
References

Supplementary Materials

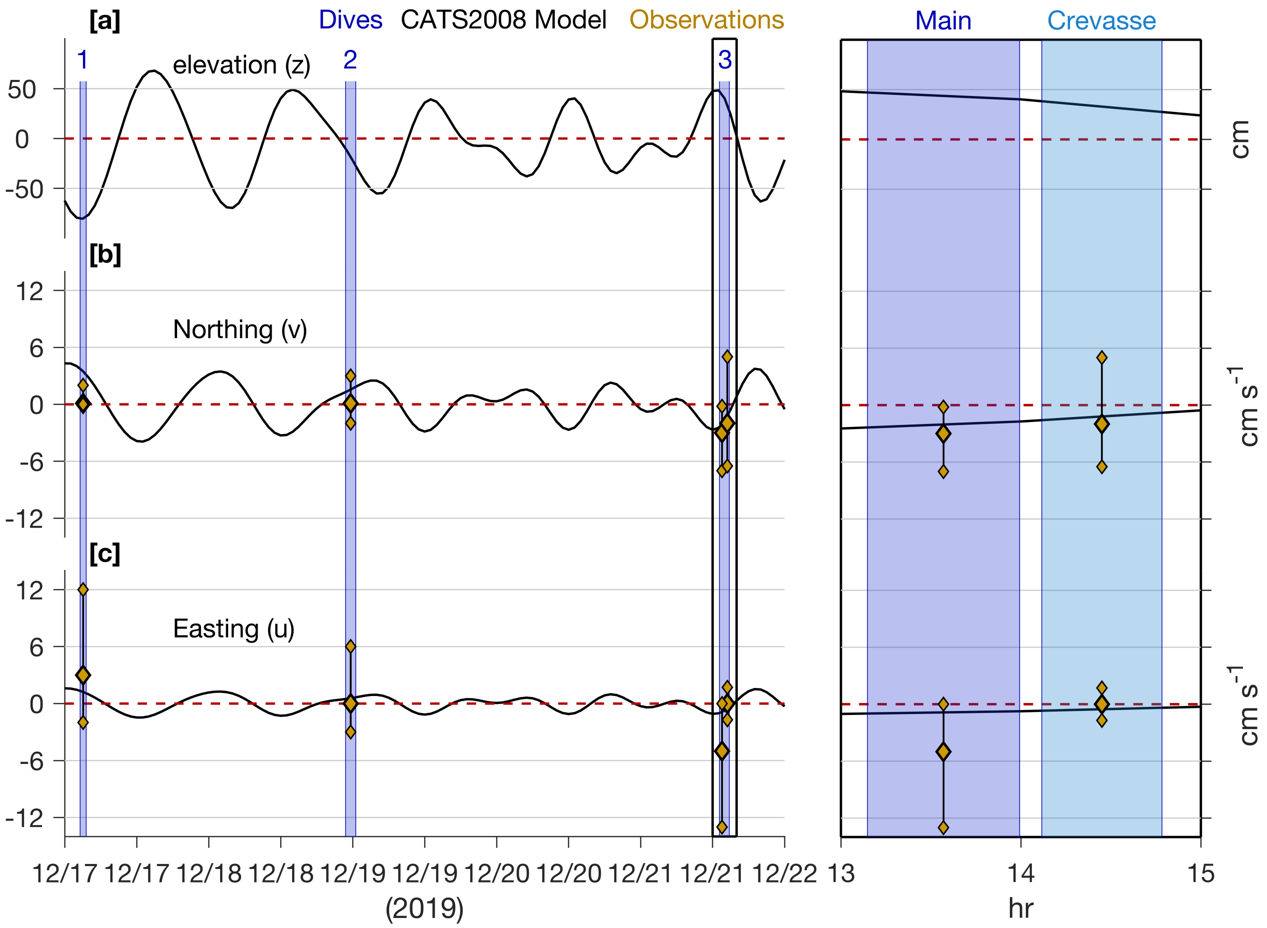

Fig. S1. Temporal position of *Icefin* dives relative to the tidal cycle. Time series of CATS2008 Model (*80*) output of the tidally-driven (a) elevation change, (b) northward or v velocity component, and (c) eastward or u velocity component at our study site. Blue shaded regions indicate the time period sampled during each *Icefin* dive and yellow diamonds show the mean (larger), minimum, and maximum measured ocean velocities. The inset focuses on dive 3, separating it into a main, outbound leg and a subsection of the return leg where *Icefin* explored the crevasse. Note that the model generally overestimates mean v velocities and underestimates u velocities relative to observations.

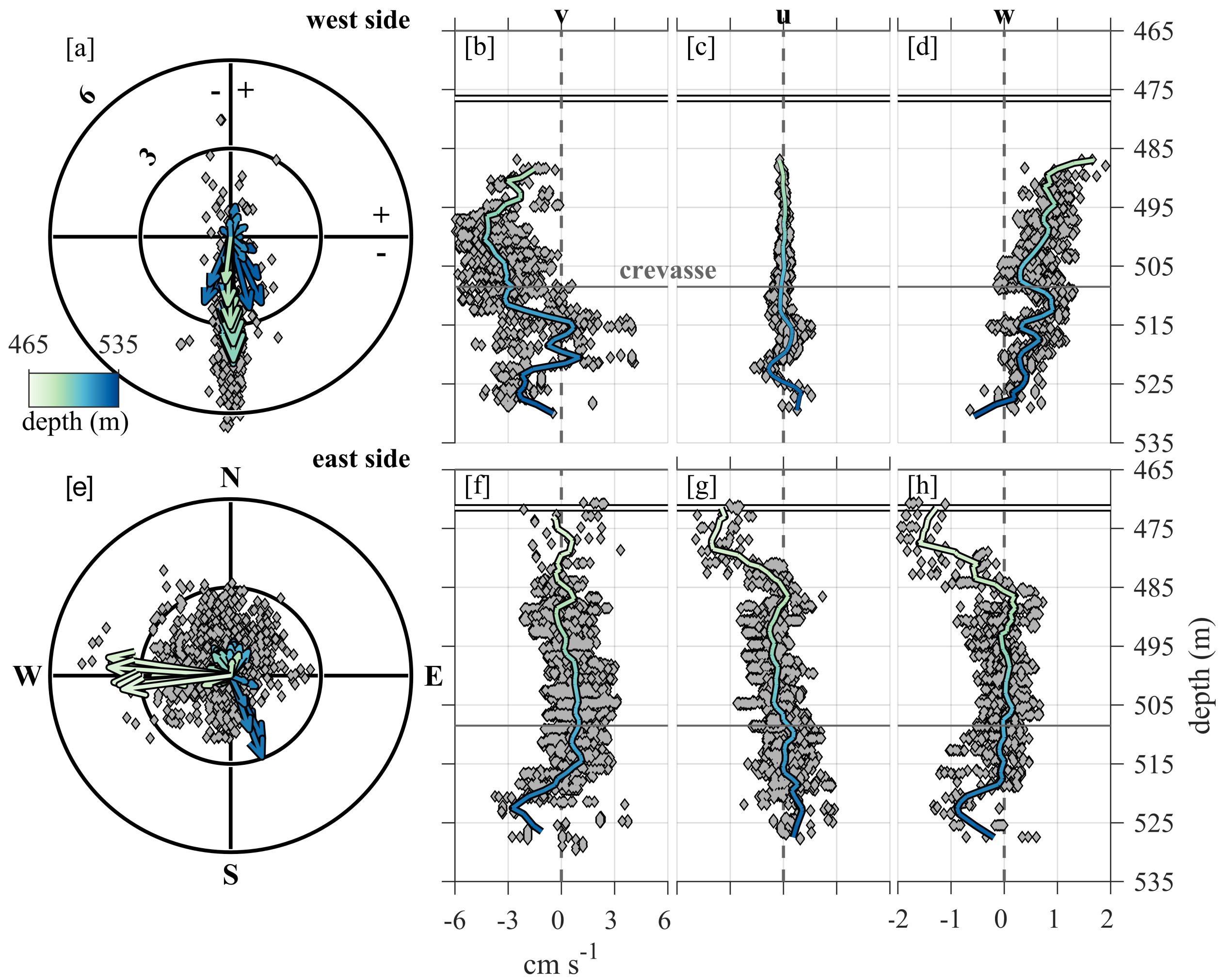

Fig. S2. Vertical profiles of ocean velocity components on either side of the crevasse. West side: (a) compass rose of u (east-west) and v (north-south) velocity components, with arrows representing average two dimensional velocities in 1 m bins. (b – d) Vertical profiles of the v, u, and w (vertical) velocity components; the heavy line is the mean at 1 m intervals, the horizontal grey lines mark the crevasse base and roof elevations, and the horizontal white line indicates the supercooling horizon. (e – h) As in (a – d), except for the east side of the crevasse. The x-axis in (c, g) is identical to (b, f). The arrows in (a, e) and the heavy lines in (b – d, f – h) are colored by depth.

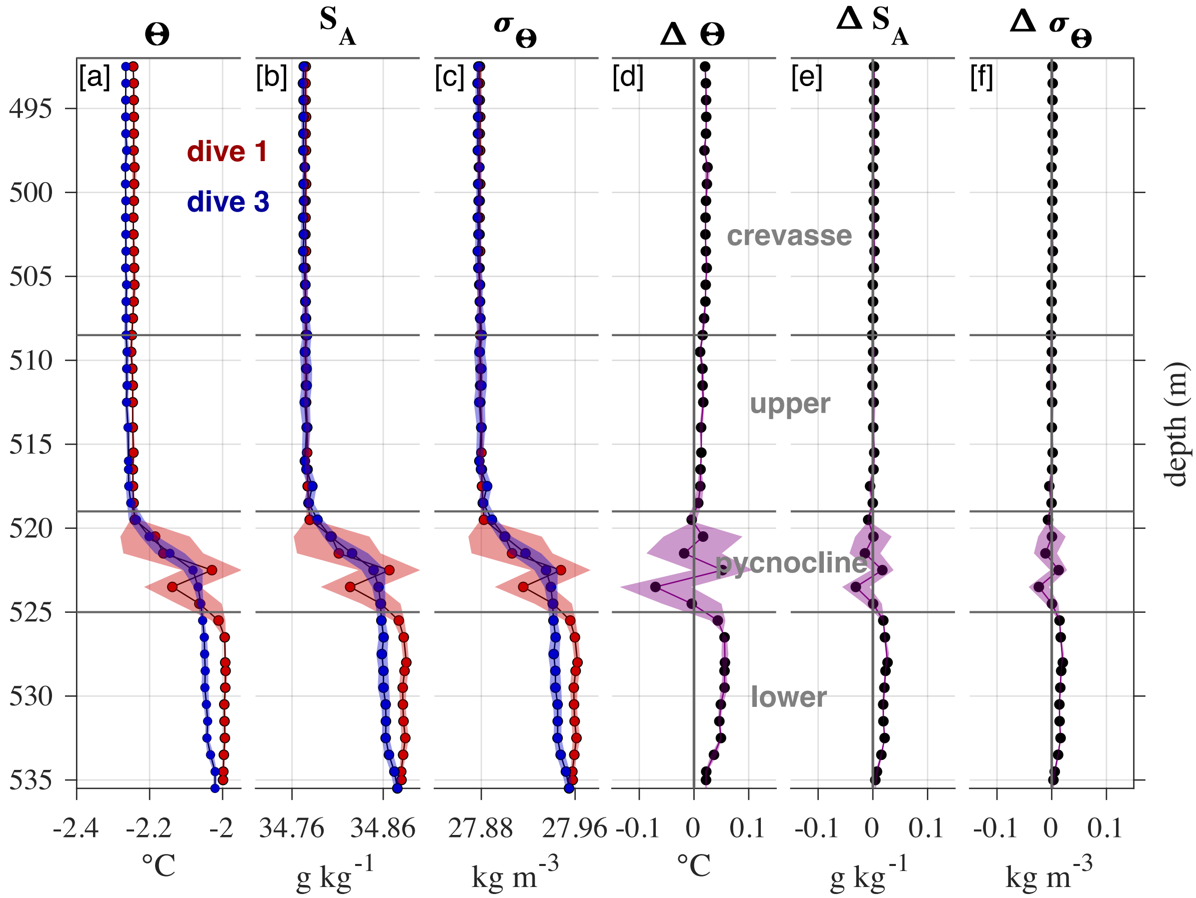

Fig. S3. Tidally-driven water column variations. Bin-averaged vertical profiles of (a) temperature, (b) salinity, and (c) density anomaly during conditions representative of spring tide (dive 1) and neap tide (dive 3) over the depth range sampled during both dives. Mean (d) temperature, (e) salinity, and (f) density anomaly differences at each depth bin between dives. Ranges around the mean reflect ± 1 standard deviation, and horizontal grey lines delineate the water column into characteristic layers. See Fig. S1 for temporal dive placement relative to the tidal cycle.

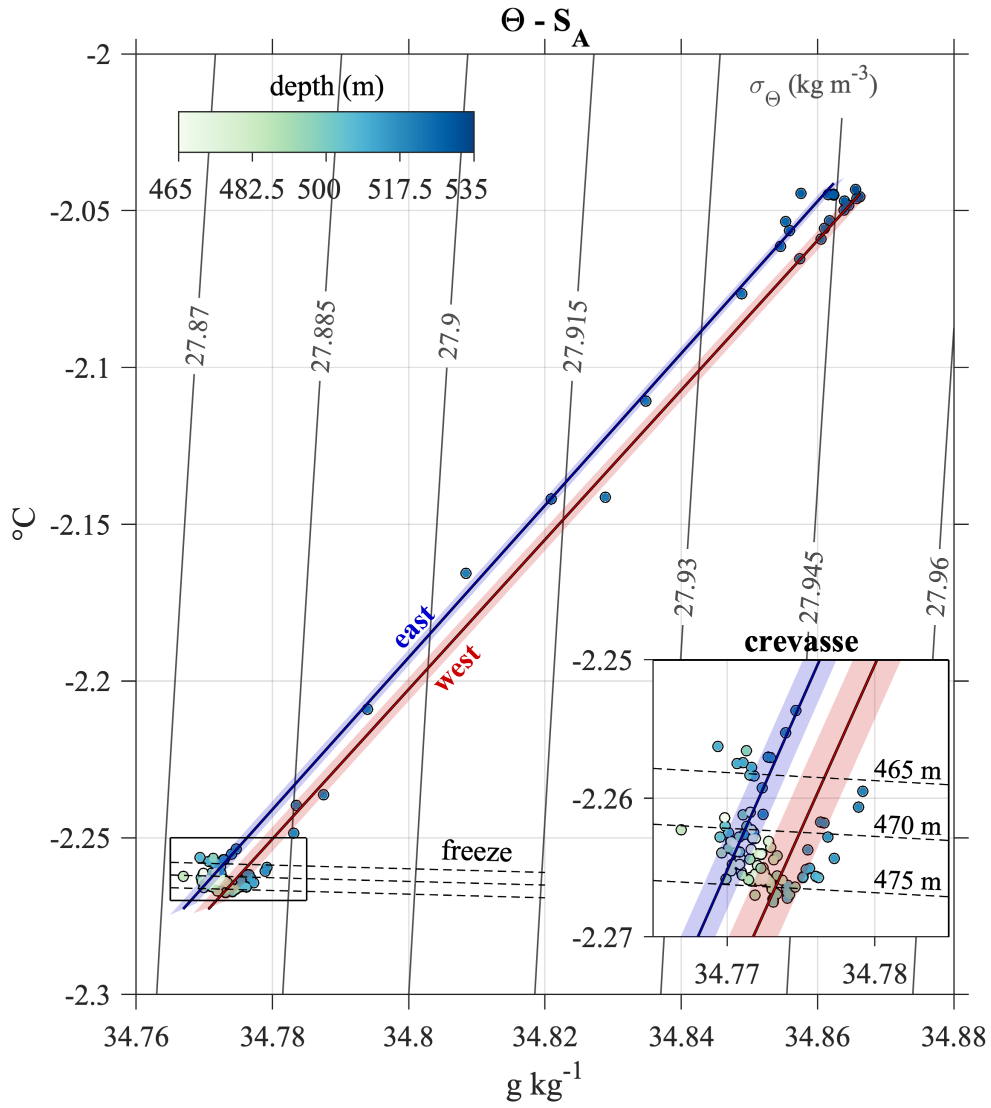

Fig. S4. Temperature-Salinity Diagram. Bin-averaged Θ and S_A values fit two distinct GMW mixing lines (Materials and Methods), one on the western (red) and one on the eastern (blue) half of the crevasse. The main figure presents data throughout the full water column and the inset shows the subset from within the crevasse; all data are colored by depth. The grey lines indicate isopycnals, the dashed lines show freezing temperatures over the observed salinity range at various depths, and the uncertainty bounds on the GMW mixing lines reflect one standard deviation ranges from bin-averaging (Materials and Methods).

Wall	depth (m)	ice morphology		C_D			depth range (m)	$z_o = \frac{1}{d}$(cm)		
		λ (cm)	d (cm)	min	mean	max		min	mean	max
West		symm. scallop		$2.70\text{x}10^{-3}$	$5.90\text{x}10^{-3}$	$9.20\text{x}10^{-3}$	<487	$\frac{1}{9.06}$	$\frac{1}{6.29}$	$\frac{1}{2.83}$
	508	48 – 63	10 – 13	law of the wall fit: $u_* = 0.32$ cm s^{-1}, $z_0 = 3.53$ cm, RMS = $8.90\text{x}10^{-3}$, N = 839 $\bar{U} = 4.25$ cm s^{-1} (only considering $U \geq 3$ cm s^{-1})						
		asymm. scallop								
	506	43 - 57								
	494	50 – 59	15 – 30							
	491	70 – 80	27 – 32							
	482	57 – 78	20 – 28	no ADCP data						
		runnel								
	476	143	148							
	475	81 – 103	82 – 85							
		reflective facie								
	466	9 - 15								
Roof		column		no ADCP data						
		72 – 140	183							
East		symm. scallop		$4.90\text{x}10^{-3}$	$7.70\text{x}10^{-3}$	$14.60\text{x}10^{-3}$	<483	$\frac{1}{9.95}$	$\frac{1}{7.99}$	$\frac{1}{5.88}$
	506	59 - 62	15 - 20	law of the wall fit: $u_* = 0.26$ cm s^{-1}, $z_0 = 2.21$ cm, RMS = $2.30\text{x}10^{-3}$, N = 70 $\bar{U} = 4.25$ cm s^{-1} (only considering $U \geq 3$ cm s^{-1})						
	503	48 - 71								
	502	70								
	501	62 - 78	22							
	494	45 - 50	13 - 17							
		runnel		*runnel data excluded because U primarily horizontal						
	503	526	212							
	502	513	210							
	501	449	203							
	494	242	210							
	481	164 - 165	100	$1\text{x}10^{-3}$	$2.30\text{x}10^{-3}$	$5.50\text{x}10^{-3}$	>483	$\frac{1}{24}$	$\frac{1}{8.90}$	$\frac{1}{2.40}$
	478	70	70	law of the wall fit: $u_* = 0.21$ cm s^{-1}, $z_0 = 4.16$ cm, RMS = $5.90\text{x}10^{-3}$, N = 23 $\bar{U} = 3.96$ cm s^{-1} (only considering $U \geq 3$ cm s^{-1})						
	474	87 - 145	25 - 50							
	-70	60 - 120	30							
		reflective facie		no ADCP data						
	466	8 - 19								

Table S1. Scales of ice morphology on crevasse walls and implications for mixing.

REFERENCES AND NOTES

1. P. Fretwell, H. D. Pritchard, D. G. Vaughan, J. L. Bamber, N. E. Barrand, R. Bell, C. Bianchi, R. G. Bingham, D. D. Blankenship, G. Casassa, G. Catania, D. Callens, H. Conway, A. J. Cook, H. F. J. Corr, D. Damaske, V. Damm, F. Ferraccioli, R. Forsberg, S. Fujita, Y. Gim, P. Gogineni, J. A. Griggs, R. C. A. Hindmarsh, P. Holmlund, J. W. Holt, R. W. Jacobel, A. Jenkins, W. Jokat, T. Jordan, E. C. King, J. Kohler, W. Krabill, M. Riger-Kusk, K. A. Langley, G. Leitchenkov, C. Leuschen, B. P. Luyendyk, K. Matsuoka, J. Mouginot, F. O. Nitsche, Y. Nogi, O. A. Nost, S. V. Popov, E. Rignot, D. M. Rippin, A. Rivera, J. Roberts, N. Ross, M. J. Siegert, A. M. Smith, D. Steinhage, M. Studinger, B. Sun, B. K. Tinto, B. C. Welch, D. Wilson, D. A. Young, C. Xiangbin, A. Zirizzotti, Bedmap2: Improved ice bed, surface and thickness datasets for Antarctica. *Cryosphere* **7**, 375–393 (2013).

2. E. Rignot, J. Mouginot, B. Scheuchl, M. Van Den Broeke, M. J. Van Wessem, M. Morlighem, Four decades of Antarctic Ice Sheet mass balance from 1979–2017. *Proc. Natl. Acad. Sci.*, **116**, 1095–1103 (2019).

3. T. K. Dupont, R. B. Alley, Assessment of the importance of ice-shelf buttressing to ice-sheet flow. *Geophys. Res. Lett.* **32**, L04503 (2005).

4. B. Miles, C. Stokes, A. Jenkins, J. Jordan, S. Jamieson, G. Gudmundsson, Intermittent structural weakening and acceleration of the Thwaites Glacier Tongue between 2000 and 2018. *J Glaciol* **66**, 485–495 (2020).

5. E. Rignot, J. Mouginot, M. Morlighem, H. Seroussi, B. Scheuchl, Widespread, rapid grounding line retreat of Pine Island, Thwaites, Smith, and Kohler glaciers, West Antarctica, from 1992 to 2011. *Geophys. Res. Lett.* **41**, 3502–3509 (2014).

6. T. A. Scambos, J. A. Bohlander, C. A. Shuman, P. Skvarca, Glacier acceleration and thinning after ice shelf collapse in the Larsen B embayment, Antarctica, *Geophys Res Lett* **31**, L18402, (2004).

7. R. M. DeConto, D. Pollard, R. B. Alley, I. Velicogna, E. Gasson, N. Gomez, S. Sadai, A. Condron, D. M. Gilford, E. L. Ashe, R. E. Kopp, D. Li, A. Dutton. The Paris Climate Agreement and future sea-level rise from Antarctica. *Nature* **593**, 83–89 (2021).

8. T. L. Edwards, S. Nowicki, B. Marzeion, R. Hock, H. Goelzer, H. Seroussi, N. C. Jourdain, D. A. Slater, F. E. Turner, C. J. Smith, C. M. McKenna, E. Simon, A. Abe-Ouchi, J. M. Gregory, E. Larour, W. H. Lipscomb, A. J. Payne, A. Shepherd, C. Agosta, P. Alexander, T. Albrecht, B. Anderson, X. Asay-Davis, A. Aschwanden, A. Barthel, A. Bliss, R. Calov, C. Chambers, N. Champollion, Y. Choi, R. Cullather, J. Cuzzone, C. Dumas, D. Felikson, X. Fettweis, K. Fujita, B. K. Galton-Fenzi, R. Gladstone, N. R. Golledge, R. Greve, T. Hatterman, M. J. Hoffman, A. Humbert, M. Huss, P. Huybrechts, W. Immerzeel, T. Kleiner, P. Kraaijenbrink, S. L. Clec'h, V. Lee, G. R. Leguy, C. M. Little, D. P. Lowry, J.-H. Malles, D. F. Martin, F. Maussion, M. Morlighem, J. F. O'Neill, I. Nias, F. Pattyn, T. Pelle, S. F. Price, A. Quiquet, V. Radić, R. Reeese, D. R. Rounce, M. Rückamp, A. Sakai, C. Shafer, N.-J. Schlegel, S. Shannon, R. S. Smith, F. Straneo, S. Sun, L. Tarasov, L. D. Trusel, J. V. Breedam, R. van de Wal, M. van den Broeke, R. Winkelmann, H. Zekollari, C. Zhao, T. Zhang, T. Zwinger, Projected land ice contributions to twenty-first-century sea level rise. *Nature* **593**, 74–82 (2021).

9. G. A. Meehl, W. M. Washington, J. M. Arblaster, A. Hu, H. Teng, C. Tebladi, B. Sanderson, G. Strand, J. B. White, Climate system response to external forcings and climate change projections in CCSM4. *J Clim* **25**, 3661–3683 (2012).

10. H. Seroussi, Y. Nakayama, E. Larour, D. Menemenlis, M. Morlighem, E. Rignot, A. Khazendar, Continued retreat of Thwaites Glacier, West Antarctica, controlled by bed topography and ocean circulation. *Geophys. Res. Lett.* **44**, 6191–6199 (2017).

11. P. Dutrieux, C. Stewart, A. Jenkins, K. W. Nicholls, H. F. J. Corr, E. Rignot, K. Steffen, Basal terraces on melting ice shelves. *Geophys. Res. Lett.* **41**, 5506–5513 (2013).

12. M. Bushuk, D. M. Holland, T. P. Stanton, A. Stern, C. Gray, Ice scallops: A laboratory investigation of the ice–water interface. *J. Fluid Mech.* **873**, 942–976 (2019).

13. S. Weady, J. Tong, A. Zidovska, L. Ristroph, Anomalous convective flows carve pinnacles and scallops in melting ice. *Phys. Rev. Lett.* **128**, 044502 (2022).

14. N. J. Wilson, C. A. Vreugdenhill, B. Gayen, E. W. Hester, Double-diffusive layer and meltwater plume effects on ice face scalloping in phase-change simulations. *Geophys. Res. Lett.* **50**, 2023GL104396 (2023).

15. D. E. Gwyther, K. Kusahara, X. S. Asay-Davis, M. S. Dinniman, B. K. Galton-Fenzi, Vertical processes and resolution impact ice shelf basal melting: A multi-model study. *Ocean Model.* **147**, 101569 (2020).

16. D. E. Gwyther, B. K. Galton-Fenzi, M. S. Dinniman, J. L. Roberts, J. R. Hunter, The effect of basal friction on melting and freezing in ice shelf-ocean models. *Ocean Model.* **95**, 38–52 (2015).

17. K. Makinson, P. G. Anker, The BAS ice-shelf hot-water drill: Design, methods and tools. *Ann. Glaciol.* **55**, 44–52 (2014).

18. A. Jenkins, P. Dutrieux, S. S. Jacobs, S. D. McPhail, J. R. Perrett, A. T. Webb, D. White, Observations beneath Pine Island Glacier in West Antarctica and implications for its retreat. *Nat. Geosci.* **3**, 468–472 (2010).

19. C. B. Begeman, S. M. Tulaczyk, O. J. Marsh, J. A. Mikucki, T. P. Stanton, T. O. Hodson, M. R. Siegfried, R. D. Powell, K. Christianson, M. A. King, Ocean stratification and low melt rates at the Ross Ice Shelf grounding zone. *J. Geophys. Res. Oceans* **123**, 7438–7452 (2018).

20. J. D. Lawrence, P. Washam, C. L. Stevens, C. L. Hulbe, H. J. Horgan, G. Dunbar, T. Calkin, C. L. Stewart, N. J. Robinson, M. R. Meister, B. Hurwitz, E. S. Quartini, D. J. Dichek, A. D. Mullen, A. Spears, B. E. Schmidt, Crevasse refreezing and signatures of retreat observed at Kamb Ice Stream grounding zone. *Nat. Geosci.* **16**, 238–243. (2023).

21. M. Minowa, S. Sugiyama, M. Ito, S. Yamane, S. Aoki, Thermohaline structure and circulation beneath the Langhovde Glacier ice shelf in East Antarctica. *Nat. Commun.* **12**, 4209 (2021).

22. R. D. Powell, M. Dawber, J. N. McInnes, A. R. Pyne, Observations of the grounding-line area at a floating glacier terminus. *Ann. Glaciol.* **22**, 217–223 (1996).

23. N. Owsianowski, C. Richter, Exploration of an ice-cliff grounding zone in Antarctica reveals frozen-on meltwater and high productivity. *Commun Earth Environ* **2**, 99 (2021).

24. B. E. Schmidt, P. Washam, P. E. D. Davis, K. W. Nicholls, D. M. Holland, J. D. Lawrence, K. L. Riverman, J. A. Smith, A. Spears, D. J. Dichek, A. D. Mullen, E. Clyne, B. Yeager, P. Anker, M. R.

Meister, B. Hurwitz, E. S. Quartini, F. E. Bryson, A. Basinski, C. Thomas, J. Wake, D. G. Vaughan, S. Anandakrishnan, E. Rignot, J. Paden, K. Makinson, Heterogeneous melting near the Thwaites Glacier grounding line. *Nature*, **614**, 471–478 (2023).

25. S. Sugiyama, T. Sawagaki, T. Fukuda, S. Aoki, Active water exchange and life near the grounding line of an Antarctic outlet glacier. *Earth Planet. Sci. Lett.* **399**, 52–60 (2014).

26. R. Reese, H. Gudmundsson, A. Levermann, R. Winkelmann, The far reach of ice-shelf thinning in Antarctica. *Nat Clim Change* **8**, 53–57 (2018).

27. G. Catania, C. Hulbe, H. Conway, Grounding-line basal melt rates determined using radar-derived internal stratigraphy. *J Glaciol* **56**, 545–554 (2010).

28. C. L. Hulbe, M. Klinger, M. Masterson, G. Catania, K. Cruikshank, A. Bugni, Tidal bending and strand cracks at the Kamb Ice Stream grounding line, West Antarctica. *J. Glaciol.* **62**, 816–824 (2016).

29. J. N. Bassis, Y. Ma, Evolution of basal crevasses links ice shelf stability to ocean forcing. *Earth Planet. Sci. Lett.* **409**, 203–211 (2015).

30. C. A. Greene, A. S. Gardner, N.-J. Schlegel, A. D. Fraser, Antarctic calving loss rivals ice-shelf thinning. *Nature* **609**, 948–953 (2022).

31. O. Orheim, J. O. Hagen, S. Østerhus, A. C. Saetrang, Glaciological and oceanographic studies on Fimbulisen dure NARE 1989/90. *Filchner Ronne Ice Shelf Programme Rep.* ***4***, 120–131 (1990).

32. S. Østerhus, O. Orheim, Oceanographic and glaciological investigations through Jutulgryta, Fimbulisen in the 1991/92 season. *Norsk Polarinst. Meddel.* **124**, 21–28 (1994).

33. M. Pedley, J. G. Paren, J. R. Potter, Localized basal freezing within George VI ice Shelf, Antarctica, *J. Glaciol.* **34**, 71–77 (1988).

34. L. Herraiz-Borreguero, J. A. Church, I. Allison, B. Peña-Molino, R. Coleman, M. Tomczak, M Craven, Basal melt, seasonal water mass transformation, ocean current variability, and deep

convection processes along the Amery Ice Shelf calving front, East Antarctica. *J. Geophys. Res. Oceans* **121**, 4946–4965 (2016).

35. K. W. Nicholls, K. Makinson, S. Østerhus, Circulation and water masses beneath the northern Ronne Ice Shelf, Antarctica. *J. Geophys. Res. Oceans* **109**, C12 (2004).

36. K. W. Nicholls, S. Østerhus, K. Makinson, T. Gammelsrød, E. Fahrbach, Ice-ocean processes over the continental shelf of the southern Weddell Sea, Antarctica: A review. *Rev. Geophys.* **47**, 3 (2009).

37. D. F. Porter, S. R. Springer, L. Padman, H. A. Fricker, K. J. Tinto, S. C. Riser, R. E. Bell, Evolution of the seasonal surface mixed layer of the Ross Sea, Antarctica, observed with autonomous profiling floats. *J. Geophys. Res. Oceans* **124**, 4934–4953 (2019).

38. C. Stevens, C. L. Hulbe, M. Brewer, C. L. Stewart, N. J. Robinson, C. Ohneiser, C. S. Jendersie, Ocean mixing and heat transport processes observed under the Ross Ice Shelf control its basal melting. *Proc. Natl. Acad. Sci.* **117**, 16799–16804 (2020).

39. A. Jenkins, The impact of melting ice on ocean waters. *J. Phys. Oceanogr.* **29**, 2370–2381 (1999).

40. C. Akhoudas, J. B. Sallée, G. Reverdin, G. Aloisi, M. Benetti, L. Vignes, M. Gelado, Ice shelf basal melt and influence on dense water outflow in the Southern Weddell Sea. *J. Geophys. Res. Oceans* **125**, e2019JC015710 (2020).

41. O. Huhn, T. Hattermann, P. E. D. Davis, E. Dunker, H. H. Hellmer, K. W. Nicholls, S. Østerhus, M. Rhein, M. Schröder, J. Sültenfuß, Basal melt and freezing rates from first noble gas samples beneath an ice shelf. *Geophys. Res. Lett.* **45**, 8455–8461 (2018).

42. P. Schlosser, R. Bayer, A. Foldvik, T. Gammelsrød, G. Rohardt, K. O. Münnich, Oxygen 18 and helium as tracers of ice shelf water and water/ice interaction in the Weddell Sea. *J. Geophys. Res. Oceans* **95**, 3253–3263 (1990).

43. I. A. Zotikov, V. S. Zagorodnov, J. V. Raikovsky, Core drilling through the Ross Ice Shelf (Antarctica) confirmed basal freezing. *Science* **207**, 1463–1465 (1980).

44. A. Khazendar, J. L. Tison, B. Stenni, M. Dini, A. Bondesan, Significant marine-ice accumulation in the ablation zone beneath an Antarctic ice shelf. *J. Glaciol.* **47**, 359–368 (2001).

45. I. J. Smith, P. J. Langhorne, T. G. Haskell, H. J. Trodahl, R. Frew, M. R. Vennell, Platelet ice and the land-fast sea ice of McMurdo Sound, Antarctica, *Ann. Glaciol.* **33**, 21–27 (2001).

46. D. E. Dempsey, P. J. Langhorne, Geometric properties of platelet ice crystals. *Cold Reg. Sci. Technol.* **78**, 1–13 (2012).

47. M. Craven, I. Allison, H. A. Fricker, R. Warner, Properties of a marine ice layer under the Amery Ice Shelf, East Antarctica. *J. Glaciol.* **55**, 717–728 (2009).

48. H. Oerter, J. Kipfstuhl, J. Determann, H. Miller, D. Wagenbach, A. Minikin, W. Graft, Evidence for basal marine ice in the Filchner–Ronne Ice Shelf. *Nature* **358**, 399–401 (1992).

49. P. R. Holland, H. F. Corr, D. G. Vaughan, A. Jenkins, P. Skvarca, Marine ice in Larsen ice shelf. *Geophys. Res. Lett.* **36**, L11604 (2009).

50. D. Jansen, A. Luckman, B. Kulessa, P. R. Holland, E. C. King, Marine ice formation in a suture zone on the Larsen C Ice Shelf and its influence on ice shelf dynamics. *J. Geophys. Res. Earth* **118**, 1628–1640 (2013).

51. D. R. MacAyeal, E. Rignot, C. L. Hulbe, Ice-shelf dynamics near the front of the Filchner-Ronne Ice Shelf, Antarctica, revealed by SAR interferometry: Model/interferogram comparison. *J. Glaciol.* **44**, 419–428 (1998).

52. E. L. Lewis, R. G. Perkin, Ice pumps and their rates. *J. Geophys. Res. Oceans* **91**, 11756–11762 (1986).

53. R. Retzlaff, C. R. Bentley, Timing of stagnation of Ice Stream C, West Antarctica, from short-pulse radar studies of buried surface crevasses. *J. Glaciol.* **39**, 553–561 (1993).

54. S. Anandakrishnan, R. B. Alley, Stagnation of ice stream C, West Antarctica by water piracy. *Geophys. Res. Lett.* **24**, 265–268 (1997).

55. M. Morlighem, C. N. Williams, E. Rignot, L. An, J. E. Arndt, J. L. Bamber, G. Catania, N. Chauché, J. A. Dowdeswell, B. Dorschel, I. Fenty, K. Hogan, I. Howat, A. Hubbard, M. Jakobsson, T. M. Jordan, K. K. Kjeldsen, R. Millan, L. Mayer, J. Mouginot, B. P. Y. Noël, C. O'Cofaigh, S. Palmer, S. Rysgaard, H. Seroussi, M. J. Siegert, P. Slabon, F. Straneo, M. R. van den Broeke, W. Weinrebe, M. Wood, K. B. Zinglersen, BedMachine v3: Complete bed topography and ocean bathymetry mapping of Greenland from multibeam echo sounding combined with mass conservation, *Geophys. Res. Lett.* **44**, 11051–11061 (2017).

56. H. J. Horgan, C. Hulbe, R. B. Alley, S. Anandakrishnan, B. Goodsell, S. Taylor-Offord, M. J. Vaughan, Poststagnation retreat of Kamb Ice Stream's grounding zone. *Geophys. Res. Lett.* **44**, 9815–9822 (2017).

57. B. Cushman-Roisin, J. M. Beckers, *Introduction to Geophysical Fluid Dynamics: Physical and Numerical Aspects* (Academic Press, 2011).

58. C. S. Watson, D. J. Quincey, J. L. Carrivick, M. W. Smith, Ice cliff dynamics in the Everest region of the Central Himalaya. *Geomorphology* **278**, 238–251 (2017).

59. A. Khazendar, A. Jenkins, A model of marine ice formation within Antarctic ice shelf rifts. *J. Geophys. Res. Oceans* **108**, 3235 (2003).

60. A. Jenkins, K. W. Nicholls, H. F. Corr, Observation and parameterization of ablation at the base of Ronne Ice Shelf, Antarctica, *J. Phys. Oceanog.* **40**, 2298–2312 (2010).

61. P.. E. D. Davis, K. W. Nicholls, Turbulence observations beneath Larsen C Ice Shelf, Antarctica, *J. Geophys. Res. Oceans* **124**, 5529–5550 (2019).

62. P. Washam, K. W. Nicholls, A. Münchow, L. Padman, Tidal modulation of buoyant flow and basal melt beneath Petermann Gletscher Ice Shelf, Greenland. *J. Geophys. Res. Oceans* **125**, e2020JC016427 (2020).

63. J. R. Jordan, P. R. Holland, A. Jenkins, M. D. Piggott, S. Kimura, Modeling ice-ocean interaction in ice-shelf crevasses. *J. Geophys. Res. Oceans* **119**, 995–1008 (2014).

64. J. Kipfstuhl, G. Dieckmann, H. Oerter, H. Hellmer, W. Graf, The origin of green icebergs in Antarctica. *J. Geophys. Res. Oceans* **97**, 20319–20324 (1992).

65. M. G. Wearing, L. A. Stevens, P. Dutrieux, J. Kingslake, Ice-shelf basal melt channels stabilized by secondary flow. *Geophys. Res. Lett.* **48**, e2021GL094872 (2021).

66. N. J. Robinson, M. J. M. Williams, P. J. Barrett, A. R. Pyne, Observations of flow and ice-ocean interaction beneath the McMurdo Ice Shelf, Antarctica, *J. Geophys. Res. Oceans* **115**, C03025 (2010).

67. S. L. Mack, M. S. Dinniman, J. M. Klinck, D. J. McGillicuddy Jr, L. Padman, Modeling ocean eddies on Antarctica's cold water continental shelves and their effects on ice shelf basal melting. *J. Geophys. Res. Oceans* **124**, 5067–5084 (2019).

68. K. J. Tinto, L. Padman, C. S. Siddoway, S. R. Springer, H. A. Fricker, I. Das, F. Caratori Tontini, D. F. Porter, N. P. Frearson, S. L. Howard, M. R. Siegfried, C. Mosbeux, M. K. Becker, C. Bertinato, A. Boghosian, N. Brady, B. L. Burton, W. Chu, S. I. Cordero, T. Dhakal, L. Dong, C. D. Gustafson, S. Keeshin, C. Locke, A. Lockett, G. O'Brien, J. J. Spergel, S. E. Starke, M. Tankersley, M. G. Wearing, R. E. Bell, Ross Ice Shelf response to climate driven by the tectonic imprint on seafloor bathymetry. *Nat. Geosci.* **12**, 441–449 (2019).

69. T. J. McDougall, P. M. Barker, Getting started with TEOS-10 and the Gibbs Seawater (GSW) oceanographic toolbox. *SCOR/IAPSO WG* **127**, 1–28 (2011).

70. I. R. Joughin, S. Tulaczyk, H. F. Engelhardt, Basal melt beneath Whillans ice stream and ice streams A and C, West Antarctica, *Ann. Glaciol.* **36**, 257–262 (2003).

71. C. D. McConnochie, R. C. Kerr, Dissolution of a sloping solid surface by turbulent compositional convection. *J. Fluid Mech.* **846**, 563–577 (2018).

72. N. J. Robinson, C. L. Stevens, M. G. McPhee, Observations of amplified roughness from crystal accretion in the sub-ice ocean boundary layer. *Geophys. Res. Lett.* **44**, 1814–1822 (2017).

73. M. G. McPhee, The effect of the oceanic boundary layer on the mean drift of pack ice: Application of a simple model. *J. Phys. Oceanogr.* **9**, 388–400 (1979).

74. R. M. Thomas, Size of scallops and ripples formed by flowing water. *Nature* **277**, 281–283 (1979).

75. D. M. Holland, A. Jenkins, Modeling thermodynamic ice–ocean interactions at the base of an ice shelf. *J. Phys. Oceanogr.* **29**, 1787–1800 (1999).

76. B. A. Kader, A. M. Yaglom, Heat and mass transfer laws for fully turbulent wall flows. *Int J Heat Mass Trans* **15**, 2329–2351 (1972).

77. S. Clark, J. Doering, Laboratory experiments on frazil-size characteristics in a counterrotating flume. *J. Hydraul. Eng.* **132**, 94–101 (2006).

78. F. Bo Pedersen, Dense bottom currents in rotating ocean. *J Hydraul Div* **106**, 1291–1308 (1980).

79. T. A. Scambos, T. M. Haran, M. A. Fahnestock, T. H. Painter, J. Bohlander, MODIS-based Mosaic of Antarctica (MOA) datasets: Continent-wide surface morphology and snow grain size. *Remote Sens. Environ.* **111**, 242–257 (2007).

80. L. Padman, M. R. Siegfried, H. A. Fricker, Ocean tide influences on the Antarctic and Greenland ice sheets. *Rev. Geophys.* **56**, 142–184 (2018).
